# Microplastics and child health: A scoping review of prenatal and early-life exposure routes and potential health risks

**DOI:** 10.1016/j.toxrep.2025.102143

**Published:** 2025-10-14

**Authors:** Sakuntala Nadarasan, Zhi Xin Phuna, Rahela Zaman, Chung Keat Tan, Normina Ahmad Bustami, Yu Bin Ho, Stephanie Julia Kosasih, Eugenie Sin Sing Tan

**Affiliations:** a Faculty of Medicine and Health Sciences, UCSI University, UCSI Heights, 1, Jalan UCSI, Taman Connaught, Wilayah Persekutuan 56000, Federal Territory of Kuala Lumpur, Malaysia; b Faculty of Medicine and Health Sciences, Universiti Putra Malaysia, Serdang, Selangor 43400, Malaysia; c Faculty of Medicine, Petra Christian University, Surabaya, Jawa Timur 60236, Indonesia; d College of Pharmacy, University of Michigan, Ann Arbor, MI, 48109, USA

**Keywords:** Fetus, Infants, Microplastics, Young children

## Abstract

Microplastics (MPs) is defined as plastic particles less than 5 mm. MPs have become a major environmental pollutant and it is originating from the breakdown of larger plastic waste. This article aims to explore MPs exposure to human health, particularly its potential effects during early developmental years. Studies reveal that MPs exposure begins via intra-uterine route, where MPs were detected in the placenta, amniotic fluid, umbilical cord, fetal membranes, and umbilical vein blood. As infants developed, the MPs exposure continued via breast milk, milk storage bags, formula, feeding bottles, and even from pacifiers. During early childhood, exposure routes can be from 3 different routes, including dermal contact (clothing, childcare products), ingestion (bottled milk, school dust, playground sand, sugar, salt), and inhalation (toy blocks, play mats, indoor air). The review also highlights the potential health risks to vital organs and systems from prenatal as well as postnatal MPs exposure in *in vivo* studies. These includes the optical, neurological, cardiovascular, pulmonary, hepatic, urinary, digestive, skeletal, lymphatic and reproductive systems. Given their small size and potential toxicity, MPs may disrupt these important developmental processes, leading to long-term health consequences. This article explores the route of MPs exposure with the potential severity and health impacts of those exposure, especially for fetuses, infants, and children. By doing so, it aims to identify any missing knowledge in this area. This research will serve as a foundation for designing future studies that identify health risks in generations exposed to MPs from a very young age.

## Graphical Abstract

**Figure:**
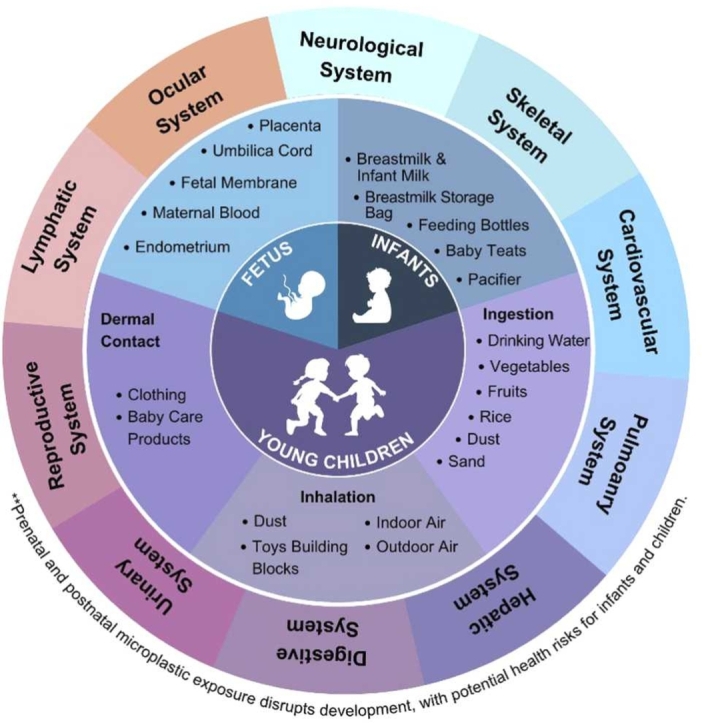

## 1 Introduction

Plastics belong to a group of synthetic materials that are made from organic polymers and some additives. Plastics were first invented in the 1950s, and since then they have revolutionized modern life due to their lightweight, strong, inexpensive, durable and corrosion-resistant properties. Hence, the global plastic production has surged from just two million tons in 1950 to over 390 million tons in 2021 [1], [2], [3]. Plastic's durability, adaptability, and extensive use in consumer products have made it indispensable, particularly in packaging, construction, textiles, consumer goods, transportation, electronics, and machinery components [2]. Nowadays, plastic has become an integral part of our lives [4]. Asia countries are projected to produce the most plastic products (50 %), followed by Europe (19 %), North America (18 %), the Middle East and Africa (7 %), and Latin America (5 %) [3], [5].

Although plastic waste and pollution can be reduced by recycling, incineration, landfill, degradation and pyrolysis, these processes are proven to contribute to the generation of microplastics (MPs). MP, as the name suggests, is a small plastic particle with a particle size smaller than 5 mm. MPs can be generated from the degradation and recycling of plastics [5]. A pilot study done by Brown et al. [6] has acknowledged the presence of MPs pollution in plastic recycling facilities in the UK, receiving 22,680 tonnes of mixed plastic waste every year. The wash water discharge from those facilities was found to contain 5.97 × 10^6^ – 1.12 × 10^8^ MP m^−3^, and the majority of MPs can be removed by filtration [7]. Several studies also supported the notion that a large number of MPs can be produced from the process of mechanical recycling [8], [9]. Mechanical recycling has gained tremendous attention due to its ability to reduce plastic waste. The implication of plastic circular economy idea, where the goal is to reduce, reuse and recycle plastic with no waste or pollution, the global discharge of MPs is expected to surge.

These ubiquitous pollutants are not only present in terrestrial ecosystems, but also in marine ecosystems and humans, harming the organisms in both land and aquatic systems. Several studies have extensively compiled the toxicity effects of MPs in both terrestrial and marine ecosystems, as well as their interactions with the microorganisms inside the ecosystems [10], [11], [12], [13]. A global review demonstrated that 60 % of 198 fish species across 24 countries were found to contain MPs [14]. Several studies also supported the presence of MPs in seafood and fishery products, which puts human health at risk when consuming these contaminated seafood products. Besides seafood, drinking water and freshwater are also found to contain substantial amounts of MPs [15], [16]. The presence of MPs in major food sources and water can eventually contaminate the food system and affect human health [2], [17], [18].

MPs can pose a significantly higher risk to human health due to their microscopic size and can be easily accumulated in the human major organs. In recent years, the topic of MPs has become a centre of attention among the scientific communities and has spurred a series of discussions following their discovery in the human body. The traces of MPs have been identified in digestive tract [19], lungs [20], blood [21], heart [22] and even bodily fluids [23], [24]. Moreover, deeper research has been done on MPs in humans following its discovery in the human placenta and fetus [25], [26], [27]. MPs were also found in amniotic fluid, meconium and infant stool, providing irrefutable evidence regarding transplacental transfer to the unborn baby [28], [29], [30]. Therefore, these exposures to MPs have raised concerns about how MPs can pose an early health risk among infants and the subsequent effects on the child’s development.

Nevertheless, there is a scarcity in the available data from human studies, particularly on the exposure of MPs in early childhood and the effect on their health due to these exposures. Infants and children are highly susceptible to adverse health risks from environmental pollutants such as MPs due to their immature immune systems. Hence, the current article aims to compile all current evidence on MPs’ exposures with a specific focus on early developmental years. This scoping review will encompass studies on identifying the primary sources of exposure and routes of exposure by the following research questions:

**RQ1:** What evidence exists on prenatal exposure to MPs through maternal transfer (placenta, amniotic fluid, cord blood) in fetus?

**RQ2:** How are infants exposed to MPs through feeding (breast milk, formula, bottles, pacifiers) and inhalation of indoor air or dust?

**RQ3:** What are the major pathways of MPs exposure in young children through diet, inhalation, and dermal contact in household and play settings?

The mechanisms by which MPs affect biological systems, and the potential short-term and long-term health consequences with special focus on fetus, infants and young children will be discussed based on the evidence from *in vitro* studies. It is hoped that this review can shed light on the extent and nature of MPs’ exposure in this particular vulnerable population.

### 1.1 Microplastic (MP) by definition and sources

**Fig. 1:**
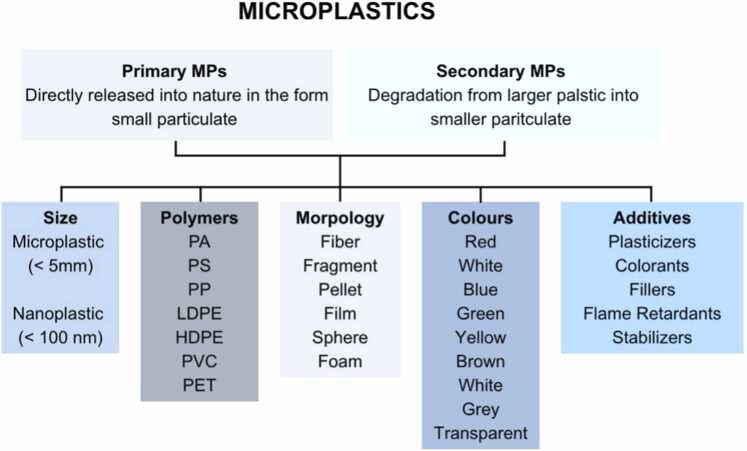
The Diversity of Microplastics in Terms of Physicochemical Attribute in terms of classification, size, polymer sources, morphology, colours and additives. *HDPE, high-density polyethylene; LDPE, low-density polyethylene; PA, polyamide, PET, polyethylene terephthalate; PP, polypropylene; PS, polystyrene; PVC, polyvinyl chloride*.

The term microplastic (MP) was first introduced in 2004 by Thompson et al., (2004) to describe the microscopic fragments of plastic debris as major oceanic plastic pollution in the United Kingdom (UK) [31]. After years of studying it, MPs are now an umbrella term for a diverse group of plastic products generally less than 5 mm in size [2]. MPs less than 1 μm or even below 100 nm are considered as nanoplastics (NPs). MPs can arise from multiple sources such as tires, textiles, cosmetics, paints and fragmentation of larger polymers [32]. Common polymers and MPs generated include high- and low-density polyethylene (PE), polyvinyl chloride (PVC), polypropylene (PP), polystyrene (PS), polyethylene terephthalate (PET), polyurethane (PU), and polyamide (nylon) (PA) (Fig. 1) [33]. They come in a varying shape, such as fibers, filaments or spheres and even irregular shapes. The toxicity of MPs depends on their size, where the smaller the size, the higher the predicted toxicity. This can be due to the greater bioavailability in the system upon ingestion and egestion of MPs [34], [35].

These MPs are produced either by direct manufacturing for a destined purpose, such as microbeads used as physical exfoliants in face cleansers, cosmetics, paint, detergents and even diapers, are often called the primary sources of MPs. There are also secondary sources, as byproducts of the degradation of the parent plastic [36], [37], [14]. These secondary MPs arise from an exhaustive list of plastic objects stretching from car tires, paint coatings, food packaging, cosmetic and personal care items, plastic pellets, synthetic textiles and so on [36], [37]. Various additives such as plasticizers, flame retardants, antioxidants and pigments can be released during the breaking of larger plastic. The process by which primary MPs degrade into secondary MPs remains unclear, but both types have detrimental effects on the environment and human health [36], [38]. Irrational mass manufacturing of plastic coupled with irresponsible waste management has already landed us in trouble bigger than we can handle. MPs pose a double threat in that they can adsorb contaminants such as persistent organic pollutants (POPs) and also act as a vector [36], [37], as well as leach harmful additives to the surrounding environment. This is because they are manufactured with various additives included to improve functional aspects or it’s quality [39].

## 2 Research methodology

This scoping review aims to study the exposure of microplastic in early developmet stage, from prenatal to young children. This review was conducted using a methodology frameworks by Arksey and O’Malley [40] and adhered to the Preferred Reporting Items for Systematic Reviews and Meta-Analyses extension for Scoping Reviews (PRISMA-ScR) to ensure rigour and transparency [41]. A scoping review checklist was incorporated in Appendix 2 to guide the process, ensuring transparency, methodological rigor, and consistency in the identification, selection, and reporting of the included studies.

### 2.1 Eligibility criteria

The inclusion criteria for this scoping review requires studies that focus on exposure of MPs from prenatal to young children, where it includes original articles that focuses on detection of MPs and estimated daily exposure of MPs. Only studies conducted in English with full-text available were included. Non-original articles, non-peer-revieewed articles, systematic reviews, reviews, case studies and conference proceedings as well as perspectives, commentary, opinion were excluded. Detection of MPs among adults or orginal articles conducted using *in vitro* and *in vivo* models were also excluded.

### 2.2 Search strategy

The database search was done by identifying relevant English papers in peer-reviewed scientific journals in Scopus and PubMed eectronic database with inclusion of research published from 2021 to 2025. The search terms includes “microplastics” AND “infants” AND “breastmilk” OR “play” OR “air” OR “house” OR “play” OR “food” OR “school” OR “drinks”. The same keywords were repeated again with “Child/Children” as “microplastics” AND “child/children” AND “breastmilk” OR “play” OR “air” OR “house” OR “play” OR “food” OR “school” OR “drinks”. All retrived articles were imported into Endnote 21 for removal of duplicates, followed by manual deduplication by importing into Microsoft Excel. To ensure accuracy and reliability, independent search was done by 2 reviewers, where the titles and abstracts were screen to identify the studies that met the selection criteria. Full-text analysis was then conducted to extract data, with any disagreements in paper selection or data extraction resolved through group discussion, and reasons for exclusion were documented. A third reviewer was involved to make a final devision when agreement could not be achieved to resolve any discrepancies.

## 3 Results

### 3.1 Articles selection and synthesis

A total of 45 studies have been selected for this stiudy (refer to Appendix 1). Among the 45 studies reviewed, 10 papers focused on prenatal exposure, where MPs were detected in critical pregnant matrices such as umbilical cord, placenta, maternal blood, and amniotic fluid, highlighting the potential for transplacental transfer of MPs into fetus. 7 studies reported on MPs in breast milk, formula milk, raw milk, conventional milk, and organic milk that suggested both natural and processed milk sources may serve as major exposure routes for infants. Another 9 papers focused on the presence of MPs in feeding bottles and breast milk storage bags. These suggested that MPs can be leached from feeding materials that may lead to substantial MPs intake in infants and young children.

Furthermore, 2 studies focus on the dermal contact of MPs. 9 studies identified MPs from indoor dust, which is of concern given the high susceptibility of infants to dust ingestion through hand-to-mouth behavior. Another 5 studies investigated the MPs in water sources, suggesting that household water can act as another possible entry point into infant diets and daily use. Additionally, 4 studies focused on atmospheric MPs, which were particularly focus on infants since inhalation during early developmental stages may represent a significant exposure pathway. Collectively, these findings illustrate that MPs can reach young children through multiple routes, from prenatal transfer, feeding practices, household environments, to the air they breathe. This study emphasizes the need for comprehensive assessments of cumulative exposure during this vulnerable life stage.

## 4 Discussion

### 4.1 Microplastic exposure from fetus to toddler

Pregnancy and infancy are both critical stages of development in humans. They are highly susceptible to trauma and environmental toxins [42]. It is predicted that environmental toxins, like MPs, could have a significant impact on the infant’s developing systems [3]. However, there is limited information on the nature and extent of these impacts on infants and young children. The risk of exposure to environmental toxins is higher in children compared to adults due to greater intake of air, food and fluids relative to body weight, crawling behaviours and short stature [3], [43]. Frequent hand-to-object behaviour and playing with electronics among toddlers are also associated with higher levels of environmental toxin exposure, such as flame retardants [44]. Additionally, children's natural curiosity and exploratory behaviours make them more susceptible to pollution, and their activities can be difficult to control, as they cannot yet differentiate between what is safe and harmful [3].

Early exposure to MPs among infants and children can occur via the placenta, breastfeeding, formula milk feeding and daily activities involving toys and textiles. These heavy exposures are especially concerning given that the immune and metabolic systems of infants are in the early stages of development. Hence, they may be unduly subjected to adverse effects from MPs [4]. This section will outline the exposure and source of MPs from fetus to toddler.

### 4.2 Intra-uterine (Prenatal) microplastic exposure in fetus

Meconium is expelled by newborns during the first or second day post partum. It is dark and thick with accumulated waste material ingested with amniotic fluid during the second and third trimesters of pregnancy [45]. Several studies identified MPs in the meconium of newborns, which indicates a possible intrauterine exposure of infants even before birth, especially during the 3rd trimester of pregnancy [46], [47], [48]. However, these findings are inconclusive due to the small sample size. Additionally, the studies are flawed because they failed to control for bias and contamination resulting from improper sample collection procedures [39], [46], [49], [50]. Moreover, a recent study published by Li et.al. (2023) found no MPs in meconium samples, suggesting that there may be no or very insignificant intrauterine transfer of MPs to the fetus [39].

However, presence of MPs in the placenta again suggested the intrauterine exposure of MPs from mother to fetus. Table 1 has summarized the preence of MPs in placental sample. In normal pregnancies, the concentration of MPs in placental samples ranged from 3.15 to 18 particles/g [24], [51], [52], [53], [54]. One study focused on detecting the MPs concentration in placenta among pregnant women with intrauterine growth restriction. It has found that as high as 40 particles/placenta of MPs can be found in IUGR pregnancies, which major polymers being PE and PS. The exposure was also reported to be negatively associated with birth outcomes such as birth weight, length, head circumference and Apgar score [51].

**Table 1:** Microplastics in Maternal Blood, Placental, Umbilical Cord, Umbilical Vein and Amniotic Fluid.

No	Sample	MPs Concentration	MPs Identified	Sample Size	Identification Method	Country	Ref.
1	Placenta	18 particles/g	PA, PU, PE, PET, PP, PVC, POM, EVA, PTFE, CPE, PC, PS, PMMA, PLA, Polysulfones	18	Agilent 8700 LDIR QCL	China	[24]
2	Placenta (normal pregnancy)	6 particles	PE, PS	30	μ-Raman	Iran	[51]
	Placenta (IUGR)	302 particles	PE, PS, PET, PP	13			
3	Placenta	40 particles	PTFE, PS, PC, ABS, PP, PE, PVC	50	μ-Raman + py-GC-MS	China	[52]
4	Placenta	3.15 particles/g	CEL, PB, PEA, PPG, PCL, PI, PNB	9	μ-Raman	China	[53]
	Cord Blood	1.1 particles/g	CEL, PB, PNB, PP	9			
5	Umbilical cord (healthy pregnancy)	82.44 mg/kg	PC, PE, PMMA, PP, PVC, PS	30	PY-GC/MS	China	[55]
	Umbilical cord (PIH)	120.7 mg/kg	PC, PE, PMMA, PP, PVC, PS	15			
6	Amniotic fluid	4.795 particles/g	PA, PU, PET, CPE, PE, PMMA, ACR, PP, FKM, PS, PVC, BR	12	Agilent 8700 LDIR	China	[54]
	Fetal membrane	6.561 particles/g	PA, PU, PET, CPE, PE, PMMA, ACR, PP, FKM	12			
	Maternal blood	8.176 particles/g	PA, PU, PET, CPE, PE, PMMA, ACR, PP, FKM, PS, BR	12			
	Placenta	4.675 particles/g	PA, PU, PET, CPE, PE, ACR, PP, FKM, PVC, BR	12			
	Umbilical vein blood	2.726 particles/g	PA, PU, PET, CPE, PE, PMMA, ACR, PP, FKM	12			
	Umbilical cord	10.397 particles/g	PA, PU, PET, CPE, PE, ACR, PP, FKM, PVC, BR	12			
7	Amniotic fluid	2.01 ± 4.19 particles/g	PE, CPE, PA, PU, PP, EVA, SBS, PET, PVC	40	Agilent 8700 LDIR	China	[56]
8	Endometrium	21 particles/100 mg	ACR, PE, PET, PP, PS, PU, PVC, BR, CPE, EAA, EVA	20	Agilent 8700 LDIR	China	[57]
9	Placenta	15 n/10 g	PVC, PP, PBS	1121	Agilent 8700 LDIR	China	[58]
10	Placenta	5.6 particles/ml	PE, PVC	10	Stereomicroscope + FTIR	Czech Republic	[59]
	Amniotic fluid	3.2 particles/ml	PE, PET, PTFE	10			

ACR, acrylate; chlorinated polymer; BR, butadine rubber; CEL, cellulose; EAA, ethylene acrylic acid; FKM, fluoroelastomers; PA, Polyamide; PB, polybutylene; PCL, polycaprolactone; PU, polyurathene, PE, polyethylene; PEA, poly(ethylene adipate); PET, polyethylene terephthalate; PI, polyimide; PLA, polylactic acid; PMMA, polymethyl methacrylate; PNB, polynorbornene; PP, polypropylene; PPG, polypropylene glycol; PS, polystyrene; PVC, polyvinyl chloride; POM, polyoxymethylene; EVA, ethylene vinyl acetate; PTFE, polytetrafluoroethylene; SBS, styrene-butadiene-styrene;

The placenta generally safeguards the fetus by creating a barrier between the matrix and the fetus [46], [49], [60], [61], [62]. The placenta is undisputedly the most vital organ for the fetus, providing nutritional support, hormonal support, oxygen supply, regulating fetal and maternal environment and safeguarding the fetus in every way [63]. Hence, presence of MPs in the placenta is reason enough for grave concern. Research using mammalian models has proven that nanopolystyrene particles can move from the maternal lungs, cross the placenta, and reach the fetal’s kidney, heart, lungs, liver, and brain during late-stage pregnancy [64]. This is in-line with an *ex vivo* placental perfusion system that showed translocation of nanopolystyrene particles from the maternal uterine circulation to the fetal circulation through the placenta [65].

Besides placenta, MPs also can be found in the umbilica cord and vein blood, where the concentration ranged from 1.1 to 10.397 particles/g as summarized in Table 1 [53], [54]. A study done by Zhang et al., (2025) have found that the concentration of MPs was higher in umbilical cord of pregnant women with pregnancy induced hypertension at 120.7 mg/kg as compared to healthy pregnant women measured 82.44 mg/kg. This 1.46 times higher of MPs concentration could be owing to the frrquent use of plastic tableware, plastic-packaged beverages and high seafood intake. Among those, high neonatal mortality and lower Apgar scores was also found to be associated with frequent plastic containers usage and takeout meals [55].

Furthermore, the presence of MPs in amniotic fluid further suggested that MPs can pass from mother to fetus via placenta (Table 1). A study done on 10 pregnant women in the University Hospital Finland showed that microplastics (between 10 and 50 μm) were detected simultaneously in both amniotic fluid and placenta in 9 out of 10 women. All 10 studied pregnant women also experienced preterm prelabour rupture of membrane, which affects the physiological singleton pregnancies [59]. Xu et al. [56] have detected MPs in 32 out of 40 amniotic fluid samples with an average abundance of 2.01 ± 4.19 particles/g. Among these MPs, PE and PVC were the most prevalent, and the MPs’ level was positively associated with seafood consumption and bottled water intake [56]. Moreover, the MPs detected were found to be negatively associated with gestational age, which highlighted the potential risk of MPs on fetus development [56].

The presence of MPs in placenta, umbilical cord and vein, as well as amniotic fluid could be owing to the MPs present in maternal blood. Placental cells are able to invade both into the tissue of the maternal uterus and blood vessels that are nearest to the implantation sites to allow the steady flow of nutrient-rich blood to perfuse the placenta. This is to ensure the maternal blood is adequately perfusing the placenta, allowing sufficient *in utero* transfer of nutrient and oxygen to infants [66]. Umbilical cord connects the fetus to the placenta in order to allow proper fetal oxygenation and nutrition [67]. Moreover, amniotic fluid is derived from maternal compartments during the early stage of pregnancy [68]. Sun et al., (2024) have detected 8.176 particles/g of MPs in the maternal blood (Table 1). The same study also found the presence of MPs in amniotic fluid, fetal membrane, placenta, umbilical vein and cord blood [54]. It can be postulated that the MPs are able to translocate from maternal blood to placenta, umbilical cord, vein, amniotic fluid and ultimately reaching to the fetus. Although there is limited studies proving the potential transfer of MPs from maternal blood to placenta or umbilica cord, MPs were proven to traverse and penetrate into placental tissue by active internalization mechanism involving micropinocytosis or phagocytosis [69]. Moreover, ingestion of PS MNPs was able to breach intestinal barrier and subsequently the maternal-fetal barrier of placenta to access in fetal circulation in Sprague dawlet rat models [70]. These studies have confirmed the intra-uterine translocation of MPs from mother to fetus via placenta route.

### 4.3 Postnatal microplastic exposure in infant

#### 4.3.1 Microplastics in breastmilk and other milk products

Breastmilk is considered the benchmark for newborns due to its wide range of macro- and micronutrients that support the nutritional requirements for infants to provide optimal growth and development [71]. Exclusive breast-feeding for the initial 6 months of the infant’s life has been highly recommended by the World Health Organization (WHO). Despite being the essential food, infants can be exposed to MPs through feeding on breast milk. There were 7 studies focusing on the detection of MPs in human breastmilk and the data are summarized in Table 2. The minimum concentration of MPs in breastmilk was detected at 0.115 particles/g and maximum at 27.9 particles/g [72], [73], [74], [75]. The presence of MPs in the breastmilk could be due to the handling practice of breastmilk. Saraluck et al., (2024) have found that the number of MPs was less likely to be detected in breast milk samples of mothers who practice good maternal hygiene, such as frequent handwashing before and after feeding, as well as using a dedicated washing product [75]. These suggest that good hygiene practices are particularly important when handling breast milk samples.

**Table 2:** Microplastics in Breastmilk, Infant Milk Powder, Conventional Milk, Organic Milk and Raw Milk.

No.	Sample	MPs Concentration	MPs Identified	Estimated Daily Intake	Sample Size	Identification Method	Country	Ref.
1	Breastmilk	20.2 particles/g	PA, PU, PE, PET, PP, PVC, POM, EVA, PTFE, CPE, PS, PMMA, PLA, Polysulfones	NS	7	Agilent 8700 LDIR QCL	China	[24]
	Infant milk	17.3 particles/g	PA, PU, PE, PET, PP, PVC, POM, EVA, PTFE, CPE, PS, PMMA, PLA		5			
2	BMSF	27.9 particles/g	PVC, PP	NS	15	μ-FTIR	Pakistan	[73]
	BMSH	5.42 particles/g	PVC, PP		8			
3	Human breast milk	0.66 particles/g	PE, PVC, PP, CPE, PVOH, PEVA, PEMA, ABS, PES, PA, PC, PS, NC	NS	34	μ-Raman	Italy	[74]
4	Human breast milk	0.115 particles/g	PP, PE, PVC, PS, PET	NS	59	μ-Raman	Thailand	[75]
5	Boxed milk powder	7 ± 3 items/100 g	PE, PET, PP, PA, PVC	410 ± 301 items/capita/year	5	FTIR	China	[76]
	Canned milk powder	4 ± 3 items/100 g	PE, PET, PP, PA, PVC		8			
	Packaging	8 items/100 g	PE, PA		3			
6	Infant milk powder	42 ± 27 MPs/100 g	PA, PE, PET, PAA, PAN, PC, SBS	49 ± 32 MPs/day (0–6 months)	30	μ-Raman	Poland	[77]
7	Conventional milk	12.40 particles/L	PMMA, PA, PU, PE. PET	0.09 – 1.61 n/kg/day	10	μ-FTIR	Romania	[78]
	Organic milk	30.83 particles/L	PMMA, PA, PU, PET	0.45 – 4.42 n/kg/day	6			
	Raw milk	29.00 particles/L	PMMA, PA	0.31 – 2.23 n/kg/day	4			

NS, not specified

CPE, chlorinated polymer; PA, Polyamide; PAA, polyacrylic acid; PAN, polyacrylonitrile; PC, polycarbonate; PU, polyurathene, PE, polyethylene; PET, polyethylene terephthalate; PMMA, polymethyl methacrylate; PNB, polynorbornene; PP, polypropylene; PPG, polypropylene glycol; PS, polystyrene; PVC, polyvinyl chloride; POM, polyoxymethylene; EVA, ethylene vinyl acetate; PTFE, polytetrafluoroethylene; NC, nitrocellulose; SBS, styrene-butadiene-styrene;

Furthermore, there are increasing reports detecting the presence of MPs in seafood and fishery product due to their ability to act as vector of compounds. The MPs in marine fish is likely due to the ingestion of plastic particles in the water or prey that previously ingested MPs [79]. The accumulation of MPs in fisery product can subsequently transferred to the humans. This is evident in Arshad et al. [73] study where healthy breastfeeding mothers from fishery community in Pakistan with daily habit of seafood consumptions was reported to contain 27.9 particles/g of MPs in breastmilk. This is 5 times higher than the MPs concentration in breastmilk from breastfeeding mothers who have no history of seafood consumption (5.42 particles/g) [73]. This findings are consistent with Ragusa et al., (2022) where possible association was found between the presence of MPs in breastmilk and mother’s eating habit that includes consumption of fish and sellfish [74]. These findings have raised awareness on the consumption habit of breastfeeding mothers as the MPs can be directly transferred to the breastmilk.

Besides breastmilk, infants can get essential nutrients from formula milk. It acts as a partial to even complete replacement for breastmilk to feed infants and children from 0 to 36 months of age [80]. MPs can detected from the formula milk and the packaging used to pack the milk powder (Table 2). There are different types of packaging for formula milk powder. Among those, boxed milk powder and canned milk powder are the most popular in the market. In Zhang et al., (2023) study on 13 brands of infant milk powder, boxed milk powder (7 ± 3 items/100 g) had higher MPs content than canned milk powder (4 ± 3 items/100 g). Inside the box, the plastic and aluminium foil packaging was found to emit around 8 items/100 g of MPs, with PE being the predominant polymer. Additionally, the same study demonstrated that MPs exposure from feeding bottle was 6.8 times higher than the milk powder, which served as the main source of MPs contamination [76]. The same study also reported that 8–14 items/day of MPs can be produced during the preparation of milk powder in milk bottles made from PE [76]. Another study conducted by Kadac-Czapska et al., (2024), reported MPs contamination in all 30 infant formula brands (average concentration of 42 ± 27 particles/100 g) purchased from the European market. The same study estimated that the average MPs intake in infants was 49 ± 32 MPs daily by considering the appropriate portion of powder per age. The MPs’ content also varied with the choice of packaging used, with three-layer composite packaging containing the highest contamination and aluminium bags containing the least [77].

Besides breastmilk and formula milk powder, MPs also have been detected in other milk including conventional, raw and organic milk (Table 2). Among these 3 types of milk, organic milk produced from cows raised on organic farm was found to contain the highest concentration of MPs at 20.83 particles/L with estimated daily intake (EDI) at 0.45–4.42 n/kg/day. On the other hand, conventional milk undergone pasteurization contained the lowest MPs concentration at 12.40 particles/L and EDI at 0.09–1.61 61 n/kg/day. PMMA and PA were the predominant MPs found across all 3 types of milk [78]. The presence of MPs in all milk samples have emphasized that the MPs contamination can raise from the basis, which is the cow’s feed and drinking water. MPs also can be released during the milk pasteurization process and the milk packaging.

#### 4.3.2 MPs in breastmilk storage bag

Furthermore, breastmilk storage bags serve as another main contributors for MPs exposure in infant. Table 3 summarized the MPs released from different breastmilk storage bag. Investigation on 6 best-selling single-use breastmilk storage bags from China found MPs in each. These storage bags contained MPs of PE, PET and PA6, at concentration of 0.32 mg/L in which the exposure was measured at 0.61–0.89 mg/day in infants based on average daily breast milk intake by them [72]. Based on the labels on the packaging, these storage bags were primarily made from PE, LPDE and PTFE. LDPE are widely used to make breastmilk storage bag as it is less susceptible to degradation and biodegradation than PE [81]. However, they are not resistance to MPs leaching despite in a single use.

**Table 3:** Microplastics in Breastmilk Storage Bag.

No	Sample	MPs Concentration	MPs Identified	Estimated Daily Intake	Sample Size	Identification Method	Country	Ref.
1	Breastmilk storage bag	0.32 mg /L	PA, PE, PET	0.61–0.89 mg/day	6	μ-Raman	China	[72]
2	PE breastmilk storage bag	4.290 particles/ml (heating at 40°C)	CPE, PA, PU, PE, PP, PET, PVC, PLA, PTFE, PMMA, EVAA	3224–5025 particles/day	18	Agilent 8700 LDIR	China	[82]
		4.673 particles/ml (heating at 80°C)	CPE, PA, PU, PE, PP, PET, PVC, PMMA, EVA, EVAA, phenol-formaldehyde resin		18			
	LDPE breastmilk storage bag	5.893 particles/ml (heating at 40°C)	CPE, PA, PU, PE, PET, PVC, PMMA, EVA, EVAA, phenol-formaldehyde resin		18			
		3.236 particles/ml (heating at 80°C)	CPE, PA, PU, PE, PP, PET, PVC, PMMA, phenol-formaldehyde resin		18			

CPE, chlorinated polymer; PA, Polyamide; PU, polyurathene, PE, polyethylene; PET, polyethylene terephthalate; PMMA, polymethyl methacrylate; PP, polypropylene; PPG, polypropylene glycol; PVC, polyvinyl chloride; EVA, ethylene vinyl acetate;

In addition, another study investigated on MPs leaching from heating both PE and LDPE made breastmilk storage bag. It was found that heating the storage bag at 80 °C could release 4.673 particles/ml of MPs from PE breastmilk storage bag and 3.236 particles/ml from LDPE storage bag. The same study compared the concentration of MPs released from storage bag after reheating at 40 °C to 80 °C but the results were insignificant [82]. This suggested that MPs can be released from storage bags made from both polymers regardless of the condition. Given the prevalent use of breast milk storage bags during breastfeeding, these results are significantly crucial as infants are constantly exposed to MPs. Even if the breast milk is free from MPs, the exposure could be coming from the the single use storage bag that puts the infants’ health at risk.

#### 4.3.3 Microplastics in feeding bottles

Besides breastmilk storage bag, there are 5 papers analyzed the presence of MPs in feeding bottles and plastic bottles in this study (Table 3). The concentration of MPs released from feeding bottles ranged from 0.625 to 393 particles/ml as summarized in Table 4 [82], [83], [84], [85], [86]. The repeated process of opening and closing the bottles can lead to the abrasion of bottles, especially the thin-necked bottles that made from lower-quality plastics, which can cause the release of MPs up to 393 particles/ml. Upon 100 cycles of opening and closing the bottles, the estimated intake of MPs per month for infant was measured at 117.3 particles, which was 10-times higher than children at 16.3 particles [83]. This has ultimately put infants at higher risks than other populations to be exposed to MPs. MPs released from PP infant feeding bottles were as high as 16,200,000 particles/L. The global exposure of MPs from this kind of feeding bottle ranged from 14,6000 – 4550,000 particles per capita per day [87]. This wide range highlights the considerable uncertainty regarding human exposure to MPs, especially in early life, and underscores the significant challenges in accurately measuring MPs.

**Table 4:** Microplastics in Feeding Bottles.

No	Sample	MPs Concentration	MPs Identified	Estimated Daily Intake	Sample Size	Identification Method	Country	Ref.
1	Feeding bottles	53 ± 9.4 – 393 ± 57.5 particles/ml	PPSU	117.3 particles/month (infant) 16.3 particles/month (children)	3	μ-FTIR and Agilent 8700 LDIR	China	[83]
	Water bottles	100 ± 23.2 – 209 particles/ml	PC, PP		4			
2	Feeding bottles	65 ± 18 items/L	PPSU, PP	2788 ± 758 items/capita/year (exposure)	3	FTIR	China	[76]
3	Plastic bottle	65.62 ± 43.28 items/L	PVC, CEL	3.09 items/kg/d (infants) 2.04 items/kg/d (children)	7	Agilent 8700 LDIR	China	[84]
	Glass bottle	87.94 ± 46.38 items/L	PVC, CEL	4.08 items/kg/d (infants) 2.70 items/kg/d (children)	3			
	Tap water	49.67 ± 21.43 items/L	PVC, silicone, PET	5.47 items/kg/d (infants) 3.61 items/kg/d (children)	NI			
4	Baby bottles	125 particles/200 ml (normal brewing)	PP	NI	NI	Agilent 8700 LDIR	China	[85]
		252.5 particles/100 ml (normal temperature with shaking)						
		288 particles/200 ml (immersed in 100°C for 10 min)						
		312 particles/200 ml (reheating for 2 min)						
5	Feeding bottles	1.68 ± 0.29 × 104	PPSU	1.63 ± 0.33 × 103 particles/kg/day	NI	FESEM + green fluorescence	China	[86]
	Food containers	3.58 ± 0.58 × 104	PP	1.17 ± 0.19 × 103 particles/kg/day	NI			
6	PPSU Feeding bottles	1.855 particles/ml (heating at 40°C)	CPE, PU, PE, PP, PET, PVC, PLA, PMMA, EVA	1040–2400 particles/day	18	Agilent 8700 LDIR	China	[82]
		2.099 particles/ml (heating at 95°C)	CPE, PU, PE, PP, PET, PVC, PMMA, EVAA		18			
	Silicone feeding bottles	1.465 particles/ml (heating at 40°C)	CPE, PU, PE, PP, PET, PVC, PMMA, EVAA		18			
		2.879 particles/ml (heating at 95°C)	CPE, PU, PE, PP, PET, PVC, PMMA, EVA, EVAA, polysulfones, phenol-formaldehyde resin		18			

CPE, chlorinated polymer; CEL, cellulose; EAA, ethylene acrylic acid; FKM, fluoroelastomers; PA, Polyamide; PU, polyurathene, PE, polyethylene; PET, polyethylene terephthalate; PI, polyimide; PLA, polylactic acid; PMMA, polymethyl methacrylate; PP, polypropylene; PPSU, polyphenylsulfone; PS, polystyrene; PVC, polyvinyl chloride; EVA, ethylene vinyl acetate; PTFE, polytetrafluoroethylene;

Other than the opening/closing cycles, the temperatures used during milk preparation can affect the amount of MPs released. In a comparison study done by Xu et al., (2023), baby bottles can release 125 particles/200 ml of MPs during normal brewing (40 °C) but the concentration increased to 252.5 particles/200 ml upon shaking. When immersing the bottles in 100 °C for 10 min, the MPs concentration increases to 288 particles and 312 particles/200 ml by reheating the milk bottles for 2 min in microwave [85]. In recent years, feeding bottles made from polyphenylsulfone (PPSU) has gained wide recognition due to its superior heat and corrosion resistance [88]. Silicone rubbeer also have been incorporated into feeding product to replicate breastfeeding [89]. However, heating feeding bottles made from both materials at 95 °C and 80 °C can release up to 2.099 and 2.879 particles/ml of MPs respectively. The predominant MPs released includes PET, PP, PE, PU and PMMA. The EDI of using these types of feeding bottles were estimated at 1040–2400 particles per day for infants [82].

With the increasing reports on toxic health effects from MP, glass bottles has been recommended to migitate the contamination of MPs especially during the process of preparing infant milk [83]. However, Li et al., (2023) have detected up to 87.94 particles/L of MPs released from glass bottle, which was higher than MPs found in PE bottles (65.62 particles/L) and tap water (49.67 particles/L) (Table 3). This could suggest the addition of MPs from water purification process before packing into bottles. At this rate, infants can expose to 3.09 and 4.08 particle/kg/day respectively when using PE and glass bottles. The EDI for children reduced almost half to 2.04 particles/kg/day (PE bottles) and 2.70 particles/kg/day (glass bottle) due to the relatively light weight of infants [84]. This findings is consistent with a recent study done on different drinks sold in France, where glass bottles was found to contain the highest concentration of MPs compared to plastic and can [90].

#### 4.3.4 Microplastics in baby teats and pacifier

Apart from feeding bottles, MPs can also be released from baby teats and pacifiers. Bottles and teats are widely used for feeding infants and young children, while pacifiers are often put in mouth by young children [91]. Steam disinfection and water wash of teats are the most frequent form of disinfection. However, disinfecting using steam can lead to degradation of the teats and produced 2.27 × 105 particles/teat, which EDI was measured at 0.66 × 106 particles by the age of 1 year [92]. Furthermore, Ekvall et al., (2023) have shown that particles in nano-sized can be released from silicone and latex pacifiers upon mechanical wear and ultraviolet (UV) radiation. Boiling the pacifier can even increase the release of nano-sized particles from silicone pacifiers [93]. The use of pacifiers, together with the daily use of feeding bottles, can ultimately exposed infants to high levels of MPs. The potential of MPs exposure route in infants were summarized in Table 5.

**Table 5:** Microplastic in Baby Teats and Pacifier.

No	Sample	MPs Concentration	MPs Identified	Estimated Daily Intake	Sample Size	Identification Method	Country	Ref.
1	Baby teats	2.2 ± 1.7 × 105 particles/teat	NS	0.66 ± 0.51 × 106 particles	NS	O-PTIR	China	[92]
2	Silicone Pacifier	20 – 25 × 106 particles/ml	NS	NS	2	ATR-FTIR	Sweden	[93]
	Latex Pacifier	10 – 35 × 106 particles/ml			1			

NS, not specified

## 5 Microplastic exposure in toddler to young children

After infants grow into toddlers and young children, the MPs exposure increases as the age increases. A probabilistic model based on the intake of eight food types, inhalation, intestinal absorption, biliary excretion and plastic-associated chemical exposure has estimated that the median intake rate of MPs is estimated at 553 particles/capita/day (184 ng/capita/day). The MPs intake can irreversibly accumulate up to 8.32 × 103 particles/capita in children until age 18 [94]. This section explains the exposure of MPs in toddlers/young children in terms of dermal absorption, inhalation and ingestion.

### 5.1 Microplastics exposure via dermal absorption

Compared to ingestion and inhalation, the absorption of MPs through skin contact has received much less attention, as the dermal barrier prevents the absorption of particles larger than 100 nm [95]. For particles to be internalized via a transdermal route, they must pass through the sublayers of the epidermis before reaching the dermal microcirculation and being transported throughout the body via the circulatory system. Thus, only MPs smaller than 100 nm (which are also known as nanoplastics, NPs) have the potential to penetrate the dermal barrier [96]. Children can be exposed to MPs through plastic packaging, skincare products like lotions and oils, and other hygiene products designed for infants. Children's skin, in comparison to adult skin, has a thinner and less effective outermost stratum corneum, making it less effective at preventing the intrusion of MPs [3].

Besides that, a study done in Sri Lanka focused on identifying the MPs in personal care products such as face wash, facial scrubs, baby products and skin cream. It was demonstrated that the tested baby products contained both low-density PE and ethylene-propylene copolymers, and they were used by almost all the parents in the study [97]. This has unarguably put the children at risk by using the products with MPs released daily. Apart from baby products, the children’s clothes are also not free from MPs and their derivatives. Herrero et al., (2023) have conducted a dermal exposure analysis of bisphenols (BPs)-containing MPs in 120 new clothes from pregnant women, newborns and toddlers. The average BPA from all clothing was dominant in both toddlers (18.7 ng/g) and newborns (5.49 ng/g). Specifically, higher BPA levels were found in the underwear/panties clothing of toddlers with median concentrations of 37.9 ng/g. With that, the dermal exposure was measured at 1.06 × 10^−9^ mg/kg/day [98]. BPA is widely used as a monomer in the manufacturing of polycarbonate plastics and epoxy resin. BPA can rapidly desorb from polymers (PA, PP, PS) and disrupt the development of the thyroid gland in children, which may affect proper neurodevelopment [99], [100]. Further study also reported the possibility of BPA leading to cancer development [101] It is worth mentioning that toddlers have a larger skin-area-to-body-weight ratio, which leads to greater dermal risk exposure to BPA. However, it should be noted that BPA is not considered as an MP, but the derivatives that leach from MPs. Table 6 shows the potential MPs exposure via skin contact in toddlers and children.

**Table 6:** Microplastics in Baby Care Products and Clothing.

No	Sample	MPs Concentration	MPs Identified	Estimated Dermal Exposure	Sample Size	Identification Method	Country	Ref.
1	Baby Care Products	NS	LDPE, EPDM	NS	3	FTIR	India	[97]
2	Clothing	366 ng/g	BPA-containing MPs	210–248 pg/kg Bw/day	77	HPLC	USA	[102]
		15 ng/g	BPS-containing MPs	8.20 – 10.10 pg/kg Bw/day				

NS, not specified

BPA, bisphenol A; EPM, ethylene propylene monomer; LDPE, low density polyethylene; MPs, microplastic

### 5.2 Microplastics exposure via inhalation

MPs with a size between 1 μm and 5 μm are likely to be deposited in the nasopharyngeal and bronchial sections of the human respiratory tract. MPs as small as 1 μm can even reach the alveoli where the gas exchanges occur [103]. A physiologically-based human respiratory tract model has been developed to evaluate the deposition and clearance of MPs overtime based on the aerodynamic diameter over 7 years. It is demonstrated that inhaled MPs with particle sizes less than 40 μm can accumulate in extrathoracic and bronchi regions, with 0.1–5 μm MPs being the primary contributors to internal burdens [104]. It is anticipated that prolonged exposure and buildup of MPs in the lungs can lead to respiratory diseases like asthma and pneumoconiosis [105].

#### 5.2.1 Microplastics in dust

The exposure through inhalation in human can happen through MPs suspended in air by various sources, including artificial fabrics, vehicle tires, construction sites, buildings and street dust [28], [106]. It can occur in both indoor and outdoor environments. There are 4 studies focusing on the exposure of MPs in infants and children from indoor as well as outdoor dust [107], [108], [109], [110]. The estimated daily intake of MPs from these dust in young children were also summarized in Table 7. Due to the higher amount of time spent in kindergarten and educational institutes, children may get the exposure of dust from there. A study done in Iran showed that the amount of MPs collected from 2 different kindergartens ranged from 121.6 ± 33.8–134.3 ± 54.4 items/mg. The EDI of inhalations rate in infants was as high as 6.050 items/kg bw/day, which was 4 times higher than EDI in children at 2.172 items/kg bw/day [108]. Another study done on indoor dust from educational institutes have deposition of MPs at 16436.67 ± 8534.06 n/kg with EDI at 0.39 ± 0.20 particles/day [109].

**Table 7:** Microplastics in Dust and Atmospheric Air.

No.	Sample	MPs Concentration	MPs Identified	Estimated Daily Intake	Sample Size	Identification Method	Country	Ref.
1	Indoor dust at homes	1257 ± 224 fibres/m^2^/day (deposition rate in high income countries)	PET, PE, PA, PU, PC, polyacrylics, PP, PS, aromatic hydrocarbon resin, alkyd resin	*Inhalation* in infants: 4.5 × 10^−5^ ± 3 × 10^−5^ mg/kg-Bw/day *Ingestion* in infants: 3.24 × 10^−2^ ± 3.14 × 10^−2^ mg/kg-Bw/day	16 countries, 74 samples	FTIR	Varied	[107]
		1268 ± 506 fibres/m^2^/day (deposition rate in middle income countries)			6 countries, 17 samples			
		3518 ± 490 fibres/m^2^/day (deposition rate in low income countries)			7 countries, 17 samples			
2	Dust in kindergarten	121.6 ± 33.8 items/mg	PE, PC, PP, PET, PA	*Inhalation:* 6.050 items/kg bw/day (infants) 2.172 items/kg bw/day (children) *Ingestion:* 0.656 items/kg bw/day (infants) 0.155 items/kg bw/day (children)	3 kindergartens (5 g of dust)	μ-FTIR and binocular microscope	Shiraz	[108]
		134.3 ± 54.4 items/mg		*Inhalation:* 5.255 items/kg bw/day (infants) 1.887 items/kg bw/day (children) Ingestion: 0.570 items/kg bw/day (infants) 0.134 items/kg bw/day (children)			Bushehr	
3	Indoor dust	16436.67 ± 8534.06 n/kg	PS, PP, PE, PET, PVC, PU	0.39 ± 0.20 particles/day (children) 0.29 ± 0.15 particles/day (adults)	60 educational institutes	SEM-EDS	Bangladesh	[109]
4	Indoor dust	1174 MPs/g	Polyester	7.4 MPs/kg bw/day (infants) 1.4 MPs/kg bw/day (toddler)	47 (apartments, hotels, universities)	FTIR and microscope	Hangzhou, China	[110]
5	Air	6.54 N·m−3 (autumn), 9.56 N·m−3 (winter), 6.23 N·m−3 (spring), 6.18 N·m−3 (summer)	PP, PE, PC, PVC, PET, PS, ABS	9.70 × 10^3^ items/year (infant) 1.69 × 10^4^ items/year (toddler) 2.87 × 10^4^ items/year (children)	117 samples	μ-Raman	China	[111]
6	Atmospheric deposition sediment	16 – 78.25 items/g	PP, PE, PET, PP	3340 – 4719 μg/kg/day	4	μ-FTIR	China	[112]
	Atmospheric deposition suspension	0.7 – 0.83 items/g	PET, PP, PE, PP, PA, acrylic, ABS		3			
7	Air	401.63 ± 71.49 particles/m^2^/day	PET, PA, PP, PE, PVC, PS, LDPE, HDPE, PU	7375.84 ± 1312.89 particles/kg-bw/day (children)	3 collection sites	ATR FTIR and SEM	India	[113]

ABS, acrylonitrile butadiene styrene; PA, Polyamide; PU, polyurathene, PE, polyethylene; PET, polyethylene terephthalate; PP, polypropylene; PS, polystyrene; PVC, polyvinyl chloride

On the other hand, dust deposition from house plays crucial role in MPs exposure among infants and children too. More importantly, the deposition rate of MPs depends on the income level of the countries. Low income countries were having the highest MPs deposition rate at 3518 fibres/m^2^/day as compared to high income countries at 1257 fibres/m^2^/day. This could be due to the lower frequency of vacuuming that was associated with higher loading of MPs. Besides that, wooden floor coatings were likely to be the source of polyvinyl-MPs contamination. It was projected that infants can received the highest dose of MPs via inhalation at 4.5 × 10^−5^ ± 3 × 10^−5^ mg/kg-Bw/day [107]. Among these studies, the estimated inhalation rates are often higher in infants as compared to children and adults. The lower body weight and shorter life-time exposure has resulted in relatively higher exposure for infants via inhalation [114].

#### 5.2.2 Microplastics in atmospheric air

While dust serves as the reservoir of MPs within indoor environments, the MPs particles are also resuspended into the air. This can create an inhalation risk that extends beyond surface contact. The detection of microplastics in air samples across different regions underscores inhalation as a major pathway of exposure. In China, Zhou et al. (2025) reported seasonal variation in airborne MPs, ranging from 6.18 to 9.56 N·m⁻³ , with common polymers including PP, PE, PC, PVC, PET, PS, and ABS. Importantly, the estimated annual intake reached 9.70 × 10 ³ items for infants, 1.69 × 10⁴ for toddlers, and 2.87 × 10⁴ for children, highlighting the vulnerability of younger populations. Complementary findings by Wu et al. (2025) showed MPs present in atmospheric deposition sediment at concentrations of 16–78.25 items/g, with corresponding estimated exposures of 3340–4719 µg/kg/day. This suggests that MPs are not only airborne but also accumulate through atmospheric settling, further contributing to human exposure via inhalation or indirect ingestion. Similarly, Prajapati et al. (2025) quantified MPs in Indian air samples at 401.63 ± 71.49 particles/m²/day, dominated by PET, PA, PP, PE, and PVC. Strikingly, the estimated daily exposure for children reached 7375.84 ± 1312.89 MPs/kg-bw/day, which is considerably higher than reported for adults, again reinforcing the heightened susceptibility of younger populations.

#### 5.2.3 Microplastics from toys and play mats

Apart from dust and air, the inhalation exposure of MPs among infants and young children can occur during play times from toy building blocks and play mats (Table 8). A study by Luo et al., (2024) has discovered that NPs and MPs can be leached from toy building bricks after play stimulation, such as assembly and disassembly of the bricks for ten times [115]. The toy industry has been highly dependent on plastic, with approximately 40 tons of plastic consumed for every 1 million US dollars in revenues [116]. Certain manufacturers have used strong plastic such as acrylonitrile butadiene styrene (ABS) in the toy bricks, but small debris particles still can form on the surface following scratching and denting [115]. Since the MPs concentration is higher indoors, inhalation of these debris or MPs in toddlers or young children during play increases the potential exposure to MPs.

**Table 8:** Microplastic in Toys and Play Mats.

No	Sample	MPs Concentration	MPs Identified	Estimated Dermal Exposure	Sample Size	Identification Method	Country	Ref.
1	Toy Building Blocks	1 × 10^3^ – 1 × 10^5^ particles/mm^2^	ABS, PA, PC	NS	50 bricks	μ-Raman	Australia	[115]
2	Play mats (control)	1.45 ± 0.35 mg/(m^2^·d)	PVC, PE, PP, PET	NS	4	Raman	China	[117]
	Play mats (Bleaching treatment)	2.40 ± 0.45 mg/(m^2^·d)						
	Play mats (UVS treatment)	9.60 ± 0.35 mg/(m^2^·d)						

NS, not specified

*ABS, acrylonitrile butadiene styrene; PA, polyamide; PC, polycarbonate; PE, polyethylene; PET, polyethylene terephthalate; PP, polypropylene; MPs, microplastics; PS, polystyrene; PVC, polyvinyl chloride*

In line with toys building bricks, the generation potential of MPs from play mats is also particularly important. Play mats are widely used in households, kindergartens and daycares. The global market of play mats reached a valuation of USD 1014.6 million in 2023, and it is expected to rise to USD 2092.08 million in 2033, indicating the global use of play mats [118]. Study from Ou et al., (2025) showed that 4 types of MPs can be released from play mat, namely PVC, PE, PP and PET. The amount of MPs released increased after sterilization treatment of the play mat with ultraviolet sterilization (UVS) and UV-bleaching [117]. Given the constant direct contact of play mats with infants and toddlers, the MPs release may pose certain health risks towards them. Sterilization practices on play mats can release an even higher number of MPs, which confer a higher risk to toddlers. Table 5 summarized the MPs released from toy building blocks and play mats. However, the EDI was not assessed in these 2 studies.

The collected papers in this study highlight a significant exposure of MPs via inhalation, particularly for infants and children who are more susceptible to respiratory deposition of MPs. This is aligned with the a recent study done by Chen et al., (2023) that detected MPs in 89.6 % of bronchoalveolar lavage fluid (BALF) in children with an average amount of 4.31 ± 2.77 items/10 ml. The inhaled MPs were predominantly smaller than 20 μm and were composed of PP, PE and PS. Moreover, the same study also acknowledged that the children’s age was negatively correlated with the MP levels in BALF, which could be owing to the underdeveloped immune system and preferential crawling and tumbling action in the indoor environment among young children [119]. This study further provides a direct biological evidence that the MPs present in the air or dust are able to internalize into the respiratory tract, which posing potential health risks among the vulnerable groups.

### 5.3 Microplastics exposure via ingestion

#### 5.3.1 Microplastics in water

Maintianing adequate intake of water are essential for the health of children [120]. Apart from water amount, the quality of water is particularly crucial. Children are increasingly exposed to microplastics (MPs) through one of the most fundamental daily necessities, drinking water. In this study, several papers have consistently shown that MPs are present not only in untreated sources but also in treated and distributed water, highlighting the inability of current purification systems to completely eliminate these particles (Table 9). In China, an investigation into the downstream Yangtze river revealed an alarmingly high MP concentrations of 4500–5130 items/L, composed mainly of PET, PE, PP, PS, PA, and PVC. Upon entering the water treatment plan, approximately 52.7 % of MPs was being removed from the water with 100 % removal for MPs above 100 μm. This lead to EDI for children to reach 30.1–49.6 items/kg/day, making this one of the highest reported exposures worldwide [121]. Such levels are concerning given the routine and unavoidable consumption of water. Children in those regions may be ingesting thousands of particles per week through this single source alone.

**Table 9:** Microplastics in Water.

No	Sample	MPs Concentration	MPs Identified	Estimated Daily Intake	Sample Size	Identification Method	Country	Ref.
1	River water	4500–5130 items/L	PET, PE, PP, PS, PA, PVC	45.5 – 70 items/kg/day (infant) 30.1 – 49.6 items/kg/day (children)	2 L of water	μ-FTIR	China	[121]
2	DWTP 1	45 MP/m^3^	PS, PA, PP, PMMA, PE	0.16–15 MPs/kg/bw/year (children)	2 L of water	μ-Raman, FTIR	Iran	[122]
	DWTP 2	20 MP/m^3^						
3	Tap water	14 ± 5.6 particles/L	HDPE, PU, PET, PLCA	1.2 particles/kg/day (children)	3 L of water	μ-Raman	South Africa	[123]
4	Raw water	7 ± 1.23 particles /L	PE, PP, PET	0.0031–0.1813 MPs/kg bw/day	NS	ATR-FTIR and µFTIR	Fiji	[124]
	Treated water	3.12 ± 0.59 particles/L		0.0410–0.0630 MPs/kg bw/day				
5	Raw water	16.13 ± 3.83 particles/L	NS	1.2 – 1.69 particles/kg/day	1 L	Rose Bengal staining and optical microscopy	Nigeria	[125]
	Treated water	10.74 ± 3.76 particles/L						
	Tap water	12.43 ± 3.92 particles/L						

NS, not specified

*DWTP, drinking water treatment plant; PA, Polyamide; PU, polyurathene, PE, polyethylene; PEA, poly(ethylene adipate); PET, polyethylene terephthalate; PI, polyimide; PLA, polylactic acid; PLCA, poly(L-lactide-co-epsilon-caprolactone; PMMA, polymethyl methacrylate; PP, polypropylene; PS, polystyrene; PVC, polyvinyl chloride;*

By contrast, findings from Iran demonstrated much lower concentrations, with water treatment plants reporting between 20 and 45 MPs/m³, leading to EDIs for children of only 0.16–15 MPs/kg/year [122]. Another research in South Africa detected 14 ± 5.6 MPs/L in tap water, which corresponded to EDI of 1.2 MPs/kg/day for children [123]. Beisdes that, studies from Fiji further emphasized the variability of MPs according to geograhic location, reporting concentrations of 7 ± 1.23 MPs/L in raw water and 3.12 ± 0.59 MPs/L in treated water. Even though the overall numbers were low, with EDIs ranging between 0.0031 and 0.1813 MPs/kg/day for untreated sources and 0.0410–0.0630 MPs/kg/day for treated water [124]. Similarly, the raw, treated, and tap water in Nigeraia were found to contain MPs, with concentrations ranging from 10.74 to 16.13 items/L and estimated intakes of 1.2–1.69 MPs/kg/day in children [125]. The collective studies revealed that the abundance of MPs are highly varied across different countries, which may be related to the local development level, economic structure and the policy developments [126].

Moreover, it is worth to mention that MPs still persists in the water system despite the water treatment and filtration. This is especially crucial for young children as they require 3 times higher water intake per unit body weight than adult due to higher body-water consposition ratio [127], [128]. Adequte water intake in children are paramount for ensuring normal physical and neurodevelopmental process [129]. Given the greater water intake, the presence of MPs in treated water put young children at even elevated risk of MPs contamination compared to adult with their under-developed immune, gastrointestinal and metabolic systems. Long-term ingestion of MPs may trigger the oxidative stress, inflammatory responses, and disruption of gut microbiota, all of which could have long-term implications for growth and development.

#### 5.3.2 Microplastics in food

Besides milk, food also represents an important contributor to MP ingestion in children. Fruits and vegetables have been shown to contain measurable amounts of MPs as summarized in Table 10. Only a limited number of studies were included in this section, as there is a vast body of literature focusing on MPs in food and beverages. The studies selected were those that were identified through the keyword searches and that reported estimated daily intake (EDI) in children, ensuring consistency and relevance to the scope of this work. In Turkey, MPs were detected in pears, tomatoes, apples, cucumbers, onions, and potatoes, with concentrations ranging from 1.5 to 3.6 particles/g. These values translated into EDIs ranged between 2.76 and 68.24 particles/kg/day for children, with apples contributing the highest intake [130]. Fruits and vegetables form a major component of diet especially for young children, not only to promote physical and mental well being, but to prevent chronic diseases such as childhood obesity [131], [132]. The presence of MPs in fruits could be due to the widespread contamination of agricultural soils and water sources, leading to uptakes by plants. Plants then act as vector for MPs and other harmful pathogens into children upon ingestion [133].

**Table 10:** Microplastics in Food and Fruits.

No	Sample	MPs Concentration	MPs Identified	Estimated Daily Intake	Sample Size	Identification Method	Country	Ref.
1	Pear	3.1 ± 1.3 particles/g	PE, PP, PET	2.76 particles/kg/day	12	ATR-FTIR	Turkey	[130]
	Tomato	3.4 ± 1.4 particles/g		16.54 particles/kg/day	12			
	Apple	3.1 ± 1.2 particles/g		68.24 particles/kg/day	12			
	Potatoes	1.5 ± 1.6 particles/g		9.39 particles/kg/day	12			
	Cucumber	3.6 ± 1.8 particles/g		13.18 particles/kg/day	12			
	Onion	2.6 ± 1.5 particles/g		11.40 particles/kg/day	12			
2	Soft Drinks	8.9 particles/L	PA, PE, PET	0.006–0.018 MP/kg/bw/day	10 (1 L each)	FTIR	Turkey	[134]
3	Seafood	8.66 – 9.04 × 104 particles/g	NS	4.48 × 102 – 48.09 × 103 particles/Kg bw/day	60 (1 g each sample)	SEM-EDX	Tunisia	[135]
4	Rice	30.3 ± 8.61 particles/100 g	PE, PET, PP, PA	0.947 particles/kg/day	10	μ-FTIR	India	[136]

NS, not specified

*PA, Polyamide; PE, polyethylene; PET, polyethylene terephthalate; PP, polypropylene*

Other commonly consumed items, such as rice, soft drinks, and seafood, have also been shown to contain MPs (Table 7). Rice in India was found to contain 30.3 ± 8.61 particles/100 g, corresponding to an EDI of nearly 1 particle/kg/day [136]. Soft drinks in Turkey were estimated to contribute 0.006–0.018 items/kg of MPs intake per day in young children [134]. Furthermore, seafood was found to contain 8.66–9.04 04 × 104 particles/g of MPs. Depending on the varied ingestion rates of different types of seafood (sardine, sea bream, mullet, sole, mussels), the EDIs of these seafood in children ranged from 4.48 × 10² to 4.8 × 10 ³ particles/kg/day [135]. This value is alarmingly high, particularly for those who regularly consumed seafood. These results also directly reflect the pressing MP contamination issue in the sea, where the ingestion rate of MPs by seafood organisms can be as high as 100 %. The ingestion can be occurred via normal feeding activity (MPs as plankton or food) or trophic transfer from prey species that already ingested MPs [137].

#### 5.3.3 Microplastics from outdoor activities

Apart food and fruits, children may ingest MPs from outdoor activities as summarized in Table 11. Active outdoor activities are important for children’s health and overall development. Playgrounds and urban parks provide a good and safer place for children to play, where they may spend many hours [138]. Upon analyzing the MPs concentration in different parts of 19 parks in Los Angeles, the MPs concentration inside the playgrounds was found to be 5 times greater than outside the playground. The MPs in the sand samples inside the playground were 72 particles/g, but the concentration reduced to 13 particles/g in the sand samples outside the playground. Among the MPs, PP and PE are most prevalent, which come from the plastic structure and products used inside the playground. Moreover, the leaves in parks were also found to contain up to 300 μm size MPs [139]. Children tend to spend more time in the playground, and the exposure to MPs can then increase via hand-to-mouth transfer and inhalation. The MPs detected in the playground were more than 100 μm, which poses a higher risk for ingestion among children.

**Table 11:** Microplastics in Playground and Kindergarten.

No	Sample		MPs Concentration	MPs Identified	Estimated Daily Intake	Sample Size	Identification Method	Country	Reference
1	Sand inside playground		72 particles/g	PP, PE, PVC, PET, PS, EVA, PMMA, PA	NS	10 spoons (2–3 g/spoon)	FTIR	USA	[139]
	Sand near playground boundaries		42 particles/g						
	Sand outside playground boundaries		13 particles/g						
	Leaves in playground		6.5 particles/cm^2^	PP, PE, PA, PET		91			
2	Dust in kindergarten	121.6 ± 33.8 items/mg		PE, PC, PP, PET, PA	0.656 items/kg bw/day (infants) 0.155 items/kg bw/day (children)	3 kindergartens (5 g of dust)	μ-FTIR and binocular microscope	Shiraz	[108]
		134.3 ± 54.4 items/mg			0.570 items/kg bw/day (infants) 0.134 items/kg bw/day (children)			Bushehr	

NS, not specified

*PA, Polyamide; PB, polybutylene; PU, polyurathene, PE, polyethylene; PET, polyethylene terephthalate; PMMA, polymethyl methacrylate; PP, polypropylene; PS, polystyrene; PVC, polyvinyl chloride; EVA, ethylene vinyl acetate;*

**Fig. 2:**
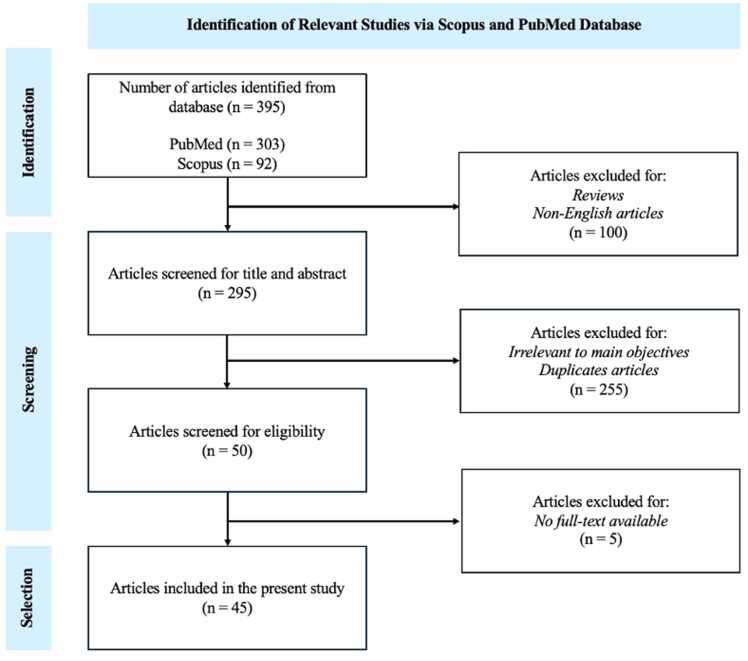
PRISMA Scoping Review Flow Diagram.

Not only that, children also may ingest the MPs from the dust in the kindergarten besides inhalation. The estimated inhalation of MPs in infants and children can be as high as 0.656 items/kg bw/day and 0.155 items/kg bw/day respectively [108]. Table 8 summarized the ingestion of MPs from the sand and leaves in playground as well as the dust from kindergarten. Although contributing at lower concentrations in intake estimation compared to dust inhalation, MPs ingestion can still represent a non-negligible source of exposure. Young children are particularly susceptible to dust ingestion due to their closer proximity to the ground dirt and indoor dust as well as oral exploratory behaviours including frequent hand-to-mouth activities and thumb-sucking [140]. The ingested MPs may be absorbed into the intestinal region and trapped in the human body throughout lifetime [141]. By using a probabilistic lifetime exposre model for children, the intake of MPs can irreversibly accumulate to 8.32 × 10^3^ particles/capita for children until age 18 [94]. The accumulation of MPs may poses greater risk for children than adults due to their immature immune and gastrointestinal system. An overview of prenatal and postnatal MPs exposure in fetus to young children has been shown in Fig. 2.

## 6 Potential health risks of early-life exposure to MPs

This area represents a significant gap in the current understanding on how exposure to MPs during pregnancy, infancy, and childhood can affect the long-term health of offsprings. In line with our focus on intra-uterine to postnatal exposure, animal studies that evaluated MPs transfer during pregnancy in rodents as well as direct exposure outcomes in neonatal and juvenile animals were considered. The exposure and potential health risk on different organ system were studied. Table 9 summarized the partial studies on the potential effects of MPs on offspring of different speices through prenatal and postnatal MPs exposure. The potential dentrimental effects were catergoried on distinct organ systems.

### 6.1 Ocular system

Maternal exposure of PS-NP in the neonatals of C57BL/6 mice were presented with delayed neural retinal development that was characterized by abnormal electroretinogram responses and enhanced level of oxidative stress in the retinas. Moreover, metabolomics analysis even revealed dysregulated level of amino acids that were critical to neuron retinal functions [142]. The exposure of MPs to the eyes could be due to the presence of those tiny polymer particles in the eyedrops or artificial drops. Eyedrops are one of the most patient compliant routes of drug delivery due to the its convenience and easy to use [143]. However, several reports from South Korea and Spain have detected the presence of MPs in eyedrops and artificial [144], [145], [146]. The detection could be due to the leaching of MPs into the eyedrops from the sotring container or bottles as they are usually made from LDPE and PP. This has unarguably put the eyes on risk particularly for those who frequently use eyedrops. Indeed, up to 1745 MPs particles were detected in the vitreous humor samples from 49 patients suffering from ocular diseases. The amount of MPs was even positively correlated with high ocular pressure and aqueous humor opacities [147]. This results demonstrated the potential toxic health effects on the eye upon the exposure to MPs.

### 6.2 Neurological system

The transfer of PS-NPs from mother to infant in mice via breastmilk can lead to neurodevelopmental alterations, including changes in brain structure and neurotransmission. This study raises the possibility that offspring that are exposed to PS-NPs through lactation may suffer from a delay in neurodevelopmental, particularly cognitive impairments [148]. An *in vitro* study was conducted using human cerebral cells and epithelial cells exposed to MPs showed cytotoxicity at the cellular level, and one of the mechanisms by which this occurred was through oxidative stress [149]. Moreover, Another study done by Yang et al., (2023) has demonstrated that postnatal exposure to PP-MPs and di-(2-ethyllhexyl)pthalic acid (DEHP) can induce cognitive and hippocampal region impairments in immature mice [150]. Simailar study also has been done in Tian et al., (2025) study where prenatal exposre to PS-NPs can result in memory impairment in the offspring with fewer neurons and more astrocytes in the hippocampus [151]. Continuous exposure can also lead to inhibition of the heat shock response, while activated unfolded protein responses ultimately induce neurotoxicity via neuronal apoptosis and neuroinflammation in immature mice [150]. Besides that, prenatal exposure of PS-NPs can lead to

Besides that, exposure to MPs can also increase the prevalence of autism spectrum disorder (ASD). Prenatal exposure to PE in C57BL/6 J and CD-1 mouse can lead to PE deposition in the brain as well as social interaction and repetitive behaviors, which serves as typical characteristic of ASD. PE exposure also resulted in increased expression of EGR-1 and ARC genes that are usually associated with neuropsychiatric disorders [152]. Furthermore, neonatal exposure of PS-MPs in newborn mice was associated with impaired microglial autophagic function and energy metabolism, disrupting the microglia-mediated synaptic pruning. This could, in turn, develop social behavioral defects in adolescent offspring [153], [154].

Moreover, cognitive and brain developmental abnormality was reported in mouse pups born to mothers who were exposed to PS-MPs during pregnancy and the lactation period. It was found that maternal exposure caused MP infiltration in pups’ brains, with modification of RNA expression and subsequent phenotypic and functional alteration of neural cell composites [155]. The PS-MPs induced molecular and functional level modifications were also studied *in-vitro* using neural cellular models. There are also reports of gender specific neurophysiological and cognitive abnormalities in offspring due to maternal exposure to MPs. The 2nd generation of female mice demonstrated a tendency to short- and long-term social recognition defects with reduced ability to socialise, and a significant increase in repetitive behavior due to mothers’ exposure to polybrominated diphenyl ethers (PBDE). The pattern of cognitive and brain developmental impairment observed was similar to that of ASD in humans [156], [157]. PBDEs belong to ubiquitous persistent POPs that are derivatives of MPs and can be found in a wide range of products such as building materials, electronics, textiles and infant products [158].

### 6.3 Skeletal & cardiovascular system

Besides brain, prenatal exposure of MPs also can accumulate in the skeleton and cardiovascular system. In the skeletal system, maternal exposure to PS-NP can result in enhanced osteoblast activity and increased bone mineral density in offspring at lower concentrations. This may suggest a beneficial or hormetic response, where low-level stressors stimulate compensatory growth. Howver, exposure of PS-NPs at 100 mg/L led to the significant reduction in the femoral growth plate thickness. The accompanying transcriptomic and metabolomic alterations also demonstrated that the fundamental cellular pathways involved in skeletal homeostasis were disrupted [159]. Chronic exposure of MPs can interfere with the bone homeostasis by inducing the inflammation and aging of bone marrow mesenchymal stem cells, which significantly affected the bone formation [160]. NPs also can promote inflammation in bone cells by stimulating the production of ROS [161]. A recent study done by Yang et al., (20250 has showed that the human bone, cartilage and intervertebral discs were presented with MPs with the highest concentration detected in intervertebral discs [162]. These ecidence provides a critical insight into the importance of MPs in skeletal health and the impacts may start as early as infant stage.

On the other hand, prenatal exposure to PA-12 particles produced overtly detrimental cardiovascular effects, including structural thinning of the left ventricular wall and significant impairments in mitochondrial function, calcium signaling, and oxidative stress defense [163]. These pathways are essential for normal cardiac growth and contractility, and their disruption during fetal development suggests a heightened risk for long-term cardiovascular dysfunction [164]. Given the centrality of mitochondrial integrity and calcium handling in maintaining myocardial energy supply and rhythm stability, these findings provide mechanistic links to potential adult-onset conditions such as cardiomyopathy, arrhythmia, and heart failure. Consistently, PE-MPs have been found in the carotid artery plaque of 150 patients asymptomatic carotid artery stenosis. The patients with MPs were also having higher risk of composite of myocardial infarction, stroke or death [165]. Patients with acute coronary syndrome were also detected with higher concentration of PE, PVC, PS and PP in the blood samples. Those particles were associated with enhanced inflammatory responses and complexity of vascular pathologies [166]. These studies reinforce the importnance of environmental contaminants exposure during critical gestation period and early life, where the consequences may only manifest in adulthood.

### 6.4 Pulmonary & hepatic system

Prenatal exposure to PS-MPs exerts profound effects on both the pulmonary and hepatic systems of offspring, with evidence showing the deposition of the MPs in the lung with collapsed alveolar and inflammation by 7th day after birth. Gene expression study further revealed the disruptions in tight junction integrity, transcriptional regulation, and transforming growth factor-beta (TGF-β) signaling pathways. The more worrying factor was that lung dysfunction and emphysematrous changes can be observed in adult offspring post 120 days of birth without any postnatal PS-MPs exposure [167]. These findings again, supported the notion that prenatal exposure of MPs can result in the accumulation of MPs in the offsprings’ lungs with compromised lung function and this impact is expected to continue until adulthood. Another study focusing on metabolomics and oxidative stess demonstrated significant imbalance in nucleic acid metabolism and amino acid profiles, alongside with elevated oxidative stress markers in the lungs of offspring after prenatally exposed to MPs. Surprisingly, melatonin, a potent antioxidant was able to improve the pulmonary dysplasia and lung function in the same offspring [168]. These results not only emphasized the on importance of redox imbalance caused by MPs, but also provided additional therapeutic options for MPs-induced damages.

Similarly, the hepatic system displayed significant susceptibility to maternal MPs exposure. Since liver is the major metabolic and detoxifying organ, it is more susceptible to toxic substances including environmental toxins [169]. The offspring of Kunming mice exposed to PS-NPs (100 nm) throughout pregnancy and lactation were experiencing reduced body and liver weight, along with impaired glycometabolism in the hepatic cells. This could be owing to the onset of oxidative stress caused by MPs with infiltration and upregulation of pro-inflammatory cells [170]. Moreover, impaired metabolic syndrome is often caused by increased uptake of fat that leads to fatty liver disease and hepatic steatosis [171]. The co-exposure of MPs with a high-fat diet during prenatal stage could exacerbate the hepatic lipid accumulation, apoptosis and hepatic inflammation in the offspring with enhanced oxidative stress in the liver [172]. Consistently, the postnatal exposure of PS-MPs and high-fat diets in juvenile zebrafish has resulted in the MPs bioaccumulation in the liver and the accumulation extend was found to be higher in the fish consuming high-fat diets priorly. The MP exposure disturbed the hepatic lipid metabolism by increasing the heaptic lipid level and ultimately lead to liver damage [173]. Taken together, these findings indicate that prenatal MPs exposure not only disrupts normal liver development but also enhanced the offspring’s vulnerability for metabolic syndrome and diet-induced liver injury.

### 6.5 Digestive & renal system

Furthermore, the gut in infants and young children is still in a developing stage with weak digestive ability and a fragile intestinal barrier. This can lower the resistance against external contamination, such as MPs [174]. Xu et al. (2023) have studied the potential risk of MPs shed from baby bottles on human intestinal cells. It was found that shaking, boiling water disinfection, and microwave heating can release MPs from baby bottles. Upon exposure to those MPs, intestinal inflammation was triggered by activating the ROS/NLRP3/Caspase-1 signalling pathway and increasing the levels of pro-inflammatory cytokines and oxidative stress [85]. Consistently, prenatal exposure of PS-NP and PP-NP can lead to surge in body weight (obesity) of the progeny by the inducing the increase of fat mass. Not only that, the lipid composition in breastmilk of the mothers mice was also altered due to long-term exposure to NPs [175]. On the other hand, the postnatal exposure of PS-MPs can result in weight loss and disrupted intestinal barrier, accompanying with dysregulated gut microbiome in the infant mice after 28 days [176]. Exposure to the same type of polymer in nano-size also can lead to similar results in juvenile zebrafish where lipid metabolism and gut microbiome were being disturbed. Combination of PS-NPs with HFD even escalate the gastrointestinal injury in juvenile zebrafish too [177].

The disturbance of gut microbiome upon exposure to MPs is evident. By using the new Toddler mucosal Artificial Colon coupled with co-culture of epithelial and mucus-secreting cells, exposure to PE can enhance the abundance of potentially harmful pathobionts such as *Dethiosulfovibrionaceae* and *Enterobacteriaceae*. However, no changes were seen in the gut barrier and permeability upon exposure to PE [178]. Furthermore, stools samples from prechool children were presented with MPs from PVC, PET, PE and PA6. These MPs were found to be negatively correlated with anti-inflammatory bacteria such as the taxa of *Lactobacillales* (order), *Rikenellaceae* (family), *Alistipes* (genus), *Streptococcaceae* (family) and *Streptococcus* (genus). *Parabacteroides* (genus) and *Lachnospiraceae_NK4A136*_group (from family *Lachnospiraceae*) were also found to be inversely proportionate with PE concentration in the study, where the decreased level of the said bacteria was closely related to the occurrence of inflammatory bowel disease (IBD) [179]. Collectively, these findings demonstrated the importance of MPs exppsure in gut barrier intergrity and gut microbiome.

Postnatal exposure to MPs in 3-weeks old Sprague Dawley rats has been shown to impair kidney health through a combination of oxidative stress, chronic gut microbiome dysbiosis [180]. Surprisingly, these effects can be improved by treating with sodium butyrate via the elevation of plasma butyric acid level and renal expression of G protein-coupled receptors 43 (GPR43) [180]. Sodium butyrate are classified as the short-cahin fatty acid salts of postbiotics that are antiproliferative for adenocarcinoma cells [181]. The same group of researchers also found that oral administration of a phenolic compound named as resveratrol also can possess the protective effects against MP-induced oxidative stress and kidney damage. The mechanism is possibly to involved the increased level of acetic acid, reduced renal expression of Olfr78 and renin-agiotension system (RAS) [182]. Resveratrol is polyphenol from class of stilbenes and known for its strong antioxidant properties in neurodegenerative, cardiovascular and renal diseases [183]. These findings suggested the potential therapeutic effects of sodium butyrate and resveratrol in MPs-induced kidney dysfunction.

### 6.6 Reproductive system

MPs/NPs have been demonstrated in some animal studies to cause reproductive dysregulations, and these particles can even be transferred to the next generation [184]. One study done on zebra fish demonstrated that MPs were able to cross the placental barrier to enter yolk sac and later bioaccumulate in organs of the developing zebra fish, including pancreas, liver, pericardium, gall bladder and even digestive tract. These MPs can induce physiological alterations such as decreased heart rate and hypoactivity in offspring, suggesting that these particles have high potential for organ toxicity [185]. In a study by Huang et al. (2022), continuous maternal administration of PS-NPs (100 nm) through drinking water from gestation until weaning has led to pronounced testicular toxicity in offspring. The weight of testis and sperm counts were reduced with disrupted seminiferous epithelim. These were found to be associated with oxidative stress with increased production of malondialdehyde and alterations in the activities of superoxide dismutase and catalase [170]. These findings underscore that nanoscale PS particles can cross biological barriers during early development and cause testicular toxicity.

Beisdes male, Dou et al. (2024) reported that prenatal exposure to larger PS-MPs (1 μm) from birth to weaning was demonstrated with delayed onset and disrupted oestrous cyclicty with impaired fertility in female mice. They were also exhibited with elevated level of serum testosterone accompanied by abnormal follicle development, ovarian steroidogenesis and ovarian inflammation. The male offspring from the MPs-exposed female mice also were demonstrated with inheritable reproductive toxicity that was associated with male germ cell proliferation, DNA methylation and histone modification [186]. Similarly, the F1 offspring from MP-exposed male mice was born with immunocompromised state where the spleen weight was increased with upregulated amount of T cells, B cells, Tregs, Th1/Th2 ratios. However, the female offsprings were shown with milder splenic immune changes. Therefore, it can be suggested that the immunotoxicity induced by PS-MPs could be passed to the next generation via sperm with gender preference [187]. These findings have emphasized on the inter- and trans-generational reproductive toxicities from MPs exposure. However, the F2 offspring from both studies were not significantly affected, highlighting a potential attenuation of toxicity across generations, possibly due to reduced transgenerational transmission.

A new perspective on reproductive toxicity was obtained where complicated pregnancy outcomes arose from an immune dysfunction related to PS-NP exposure. Hu (2021) demonstrated that peri-implantation exposure to PS-MPs (10 μm) by intraperitoneal injection markedly increased embryo resorption rates in an allogeneic mating model. The observed vascular remodeling defects, including reduced uterine arteriole number and diameter, suggested impaired maternal-fetal blood supply. Immune dysregulation was also evident, with reduced percentage of decidual natural killer (NK) cells, increased placental helper T cells, and a macrophage shift towards an M2 phenotype. These collectively drove an immunosuppressive cytokine profile in the exposed mice that subsequently led to greater chance of embryo resorption and spontaneous abortion rate in pregnant mice [188]

**Fig. 3:**
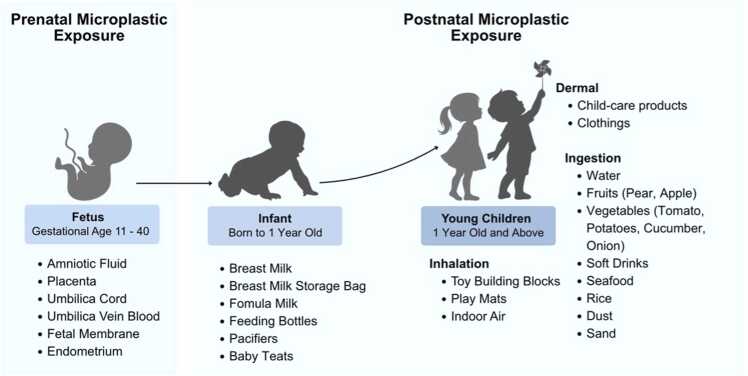
Potential Exposure of MPs from Infants to Young Children.

**Fig. 4:**
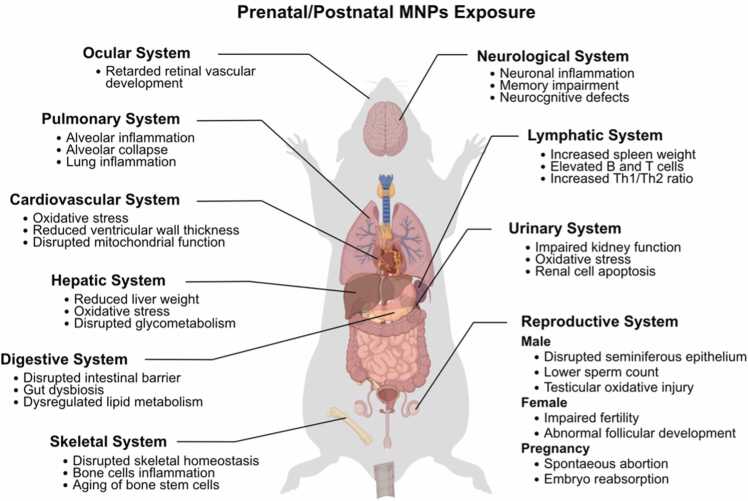
In Vivo Studies on the Potential Toxic Effects from Prenatal and Postnatal Exposure to MPs.

The collective evidence and studies have demonstrated that maternal and early-life exposure to MPs and NPs can exert profound and multi-systemic effects in animal models. The potential effects span across different biological system such as the ocular, neurological, skeletal, cardiovascular, pulmonary, hepatic, digestive, urinary, reproductive, and immune systems (as summarized in Fig. 3 and Table 12). MPs/NPs can disrupt the normal development and physiology through diverse but interlinked mechanisms. These include oxidative stress, endocrine disruption, immune modulation, metabolic dysregulation, vascular remodeling, and altered cell fate determination. These findings also indicate that MPs/NPs are not only inert contaminants but biologically active agents that are capable to perturb the developmental and physiological processes across multiple organ systems, particulary during the critical development stage Fig. 4.

**Table 12:** The In Vivo Studies on Prenatal and Postnatal MPs Exposure.

No	Exposure	Species	MPs/NP	Size	Exposure Method and Duration	Outcomes	Ref.
Ocular System							
1	Prenatal	C57BL/6 mice	PS-NPs	100 nm	The male and female mice were randomly assigned to: 1. Control (regular water) 2. Exposure groups (water dosed with 10 mg L−1 PS-NPs) Exposure was given from the day of mating.	Exposure to PS-NPs retarded retinal vascular development, while abnormal electroretinogram (ERG) responses and an increased level of oxidative stress were also observed in the retina of the progeny mice. Retinal development and function in progeny was affected.	[142]
Neurological System							
1	Prenatal	Sprague Dawley rats	PS-NPs	100 nm	Dams were orally administrated with PS-NPs at a dose of 2.5 mg/kg/day for the whole lactation period.	Offspring hippocampal region showed fewer neurons, more astrocytes, and more excitatory neurons 1(ExN1), that could contribute to memory impairment.	[151]
2	Postnatal	ICR mice	PP-MPs	7–20 μm	Three-weeks mice were fed with di-(2-ethylhexyl) phthalic acid (DEHP) and MPs in combination in different concentrations.	Exposure to PP-MPs and/or DEHP induceded hippocampal CA3 region impairment and neurocognitive defects in immature mice.	[150]
3	Prenatal	Sprague Dawley rats	PS-NPs	56.71 nm	The pregnant rats were randomly allocated into four groups: 1. Control group 2. Pregnancy group receiving PS-NPs 3. Lactation group receiving PS-NPs 4. Pregnancy and lactation group receiving PS-NPs	Maternal exposure: No significant impact on offspring brain metabolism Prenatal exposure: diminished cortical thickness and heightened cortical cell proliferation in offsprings with disordered neocortical migration, typified by escalated superficial layer neurons proliferation and reduced deep layer neurons populations.	[154]
4	Prenatal & Postnatal	C57BL/6 J mice	PE-MPs	10–20 μm	*Prenatal model:* Parent mice were fed PE for 2 weeks during pregnancy and behavioural studies were done on 5–6 weeks old offspring. *Post-weaning model:* 4-week-old C57BL/6 J mice were fed PE for 2 weeks *Puberty model:* 6-week-old puberty model mice were fed PE for 2 weeks. *Adult model:* 6-week-old C57BL/6 J mice were fed PE for 12 weeks.	PE exposure induced ASD-like behavior in the post-weaning period, puberty, and in adult model mice. Exposure to PE leads to impaired social interaction and repetitive behaviors, disturbance of metabolites and gene expression with dysregulated gut microbiome in mice model.	[152]
5	Prenatal	C57BL/6 J mice	PS-NPs	50–500 nm	Jelly cubes containing PS-NP particles were maternally administered once a day from embryonic day 8 until 2 weeks after birth	Maternal administration of PSNP during gestation and lactating periods altered the functioning of neuronal stem cells, neural cell compositions, and brain histology in progeny.	[155]
Skeletal System							
1	Maternal	C57BL/6 J mice	PS-NP	100 nm	Exposure groups were given water with 10 mg/L or 100 mg/L PS-NPs starting on the mating day.	Maternal exposure to PS-NPs (10 mg/L) via drinking water increased osteoblast numbers, bone mineral density, and bone content in offspring mice. Multi-omics analysis further showed that both low (10 mg/L) and high (100 mg/L) exposures disrupted gene expression and metabolic regulation in the offspring skeletal system.	[159]
Cardiovascular System							
1	Prenatal	Sprague Dawley rats	PA−12	6 nm −8 μm	Pregnant dams were exposed to PA−12 particles (10.46 ± 0.40 mg/m^3^) starting on gestational day 4 for 4 h/day, 5d/week, until gestational day 19	Exposed neonates demonstrated decreased relative left ventricle wall thickness with disrupted mitochondrial function, calcium handling, and defence against oxidative species.	[163]
Pulmonary System							
1	Prenatal	Sprague-Dawley rats	PS-MPs	5 μm	Dams were randomly assigned to three groups: 1. NC group (drinking water without PS-MPs during pregnancy) 2. MPL group (low-dose PS-MPs at 100 μg/L via drinking water throughout pregnancy) 3. MPH group (high-dose PS-MPs at 1000 μg/L in drinking water during pregnancy	By 7th day, PS-MPs deposits, alveolar collapse, and inflammation were observed in lung tissue. Gene expression analysis showed disruptions in tight junctions, transcriptional regulation, and transforming growth factor-beta (TGF-β) pathways. By day 120, lung dysfunction and structural changes, consistent with emphysema were observed.	[167]
2	Prenatal	Sprague Dawley rats	PS-MPs	5 μm	For metabolomics study: Dams were divided into two groups: 1. Control group (PS-MPs free drinking water) 2. MPs group (drinking water containing PS-MPs at a concentration of 1000 μgL) For Oxidative stress study: Dams were divided into three groups: 1. Control group 2. MPL group (PS-MPs at 100 g/L in drinking water) 3. MPH group (PS-MPs at1000 μg/L in drinking water)	Prenatal exposure to PS-MPs led to significantly increased oxidative stress in lung tissues, characterized by notable imbalances in nucleic acid metabolism and altered profiles of specific amino acids.	[168]
Hepatic System							
1	Prenatal	Kunming mice	PS-NPs	100 nm	Pregnant mice were continuously administered PS-NPs dispersed in drinking water at doses of 0, 0.1, 1 and 10 mg/L from gestational 0 to weaning on postnatal day 21	Maternal PS-NPs exposure in pregnancy and lactation resulted in a decline in birth and postnatal body weight in offspring mice. Reduced liver weight, triggered oxidative stress, caused inflammatory cell infiltration, up-regulated proinflammatory cytokine expression, and disturbed glycometabolism were observed in the liver of male offspring mice.	[170]
2	Prenatal	Sprague Dawley rats	MPs	5 μm	Pregnant rats were divided into the following: 1. HFD-L (HFD + microplastics, 5 µm, 100 μg/L) 2. HFD-H (HFD + microplastics, 5 µm, 1000 μg/L) 3. NCD-L (NCD + microplastics, 5 µm, 100 μg/L) 4. NCD-H (NCD + microplastics, 5 µm, 1000 μg/L)	Increased hepatic lipid accumulation, cellular apoptosis with increased lipid peroxidation markers was observed in HFD-L and HFD-H groups	[172]
3	Postnatal	Juvenile zebrafish	PS-MPs	5–50 μm	Juvenile zebrafish were subjected to either normal diet or high fat diet daily for 4 weeks. After two weeks of feeding, zebrafish were transferred to glass tanks containing either culture water or culture water containing MPs.	MP bioaccumulation was higher in the high-fat diet group, where MP aggravated hepatic lipid accumulation, liver injury, and disrupted lipid metabolism and energy homeostasis.	[173]
Digestive System							
1	Postnatal	C57-BL/6 infant mice	PS-MPs	5 μm	Infant mice were orally administrated with PS-MPs for 28 days after domestication	Accumulation of PS-MPs, weight loss, disrupted intestinal barrier, abnormal hepatic lipid metabolism, and dysregulated gut microbiome was observed in the infant mice.	[176]
2	Prenatal	C57BL/6 J mice	PS-NP, PP-NP	0–500 μg	PS and PP NP (0–500 µg) containing agarose jelly cubes were fed to pregnant female mice during pregnancy and lactation.	Maternal ingestion of NPs increase the body weight of progeny by the elevation of fat mass and PS-NP induced obesity-like microbial distribution in the gut. The lipid composition in breast milk and progeny plasma was altered.	[175]
3	Postnatal	Juvenile zebrafish	PS-NP	5–50 μm	Juvenile zebrafish was exposed to 1000 μg/L PS-NPs and a high-fat diet (HFD)	PS-NPs perturbed the lipid metabolism and gut microbiota stability in zebrafish. Combined effects of PS-NPs and HFD resulted in gastrointestinal injury in juvenile zebrafish	[177]
Urinary System							
1	Postnatal	Sprague Dawley rats	MP	5 μm	Young male offspring (3 weeks old)were randomly assigned to four groups: 1. Control (tap water) 2. Low-dose MP (tap water containing 1 mg/L MP) 3. High-dose MP (tap water containing 10 mg/L MP) 4. High-dose MP with sodium butyrate (tap water containing 10 mg/L MP and sodium butyrate)	High-dose MP exposure impaired kidney function and increased blood pressure, which were alleviated by sodium butyrate through reduced oxidative stress, modulation of gut microbiota, increased plasma butyric acid levels, and enhanced renal short chain fatty acid-sensing G protein-coupled receptor 43 expression	[180]
2	Postnatal	Sprague Dawley rats	PS-MP	5 μm	After weaning, three-week-old male SD rats were randomly allocated into four groups: 1. Control group receiving distilled water (CN) 2. Low-dose group receiving 1 mg/L (MPL) 3. High-dose group receiving 10 mg/L MP (MPH) 4. High-dose group receiving MP with resveratrol (50 mg/L; MPHR).	High dose MPs led to oxidative stress, elevated blood pressure and increased creatinine level in the kidney of the offsprings. The detrimental effects could be alleviated by resveratrol via targeting the oxidative stress, gut microbiota and renal artery stenosis	[182]
3	Postnatal	Sprague Dawley rats	PS-MP	1 μm	3 weeks old rats were divided into: 1. Control (tap water) 2. 2.0 mg/kg/d PS-MPs exposure group 3. NAC group 4. Sal group	PS-MP exposure induced kidney lesions by disrupting BUN, CRE, and pro-inflammatory cytokines (IL−1β, IL−6, TNF-α). It also triggered ER stress, oxidative stress, and inflammation, leading to renal cell apoptosis, evidenced by increased TUNEL-positive cells and upregulation of apoptosis-related genes (Bcl−2, Bax, Caspase−12, Caspase−9, Caspase−3) with elevated Caspase−12 IHC scores.	[189]
Reproductive System							
1	Prenatal Postnatal	Kunming mice	PS-NPs	100 nm	Pregnant mice were continuously administered PS-NPs dispersed in drinking water at doses of 0, 0.1, 1 and 10 mg/L from gestational day 0 to weaning on postnatal day 21	Pre- and postnatal PS-NPs exposure diminished testis weight, disrupted seminiferous epithelium and decreased sperm count in mouse offspring. Moreover, PS-NPs induced testicular oxidative injury, as presented by increased malondialdehyde generation and altered superoxide dismutase and catalase activities in the testis of offspring mice.	[170]
2	Prenatal	ICR mice	PS-MP	1 μm	Newborn female ICR mice were randomly divided into 3 groups: 1. Control 2. MP-high (MP-H, 2.4E+6 particles/g bodyweight per day) 3. MP-low (MP-L, 2.4E+3 particles/g bodyweight per day). The grouped mice were exposed to PS-MPs by gavage daily from birth to weaning (the lactation period). F1 was generated from the mating of exposed F0 female adults (Control group and MP-H group) and wild male mice (10 weeks old). F2 was generated from the mating of F1 female adult (Control group and MP-H group) and wild male mice (10 weeks old).	PS-MPs delayed puberty onset and disrupted oestrous cyclicity with impair fertility, increased serum testosterone level and abnormal follicular development in F0 female mice. Maternal exposure to PS-MPs during lactation exerted adverse effects on spermatogenesis in F1 male offspring, while F2 male offspring remained unaffected.	[186]
3	Prenatal	C57BL/6-mated BALB/c mice	PS-MPs	10 μm	Pregnant mice were divided into 1. Control group 2. MP-exposed group (intraperitoneally injected with PS-MP particles at a dose of 250 μg in a 200 μL saline solution on days 5.5 and 7.5 of gestation)	In the allogeneic mating murine model, exposure to 10 µm PS-MPs during the peri-implantation period increased embryo resorption rates. Uterine arterioles showed reduced number and diameter, potentially limiting blood supply. Immune alterations included a decrease in decidual natural killer cells, an increase in placental helper T cells, and a marked shift in macrophage polarization towards the M2 subtype. Cytokine secretion also shifted towards an immunosuppressive profile.	[188]
Lymphatic System							
1	Prenatal	ICR mice	PS-MP	1 μm	New born male mice (F0) were divided into three groups: 1. Control group 2. MPs low does group (MP-L) 3. MPs high does group (MP-H) From birth until weaning (21 days), pups were gavaged daily with either MPs at designated concentrations (experimental groups) or distilled water (control). Once matured, F0 males from the control and MP-H groups were mated with wild-type ICR females at a 1:2 ratio to produce the F1 generation. Adult F1 males were subsequently mated with wild-type ICR females to generate the F2 generation	PS-MP exposure increased spleen weight and elevated B cell and regulatory T cell (Treg) numbers in mice, regardless of dosage. In F1 male offspring, spleens were enlarged with higher numbers of B cells, T helper (Th) cells, and Tregs, alongside increased Th17/Treg and Th1/Th2 ratios, indicating a pro-inflammatory state. By contrast, F1 females showed milder splenic immune changes. In the F2 generation, spleen morphology and immune cell populations were minimally affected.	[187]

ASD, autism spectrum disorder; BUN, blood urea nitrogen; CRE, creatinine; ER, endoplasmic reticulum; IL-1β, interleukin-1 beta; IL-6, interleukin-6; PA, polyamide; PE, polyethylene; PS, polystyrene; TNF-α, tumour necrosis factor-alpha.

## 7 From rodent offspring to human infants

The evidence from animal studies clearly demonstrates that both prenatal and postnatal exposure to MPs and NPs can lead to potential multi-organ toxicity and long-term health impairments in offspring. In mice and rats, maternal ingestion of MPs/NPs during pregnancy or lactation has been shown to impair organ development (reina, brain, liver, lung, kidney, testis, and ovary), disrupt endocrine and immune function, and induce oxidative stress and the subsequent inflammation. These alterations often persist beyond weaning and manifest through different generations. When these findings are considered in the context of infants and young children, the parallels are concerning. Similar to rodent offspring, human infants are highly vulnerable due to their rapid growth, high metabolic demands, and immature detoxification systems. Moreover, multiple studies have documented that infants are exposed to MPs through breastfeeding, formula prepared in plastic bottles, ingestion of indoor dust, and even *in utero* via placental transfer. This mirrors the prenatal and postnatal exposure routes modeled in animal experiments.

Thus, the biological mechanisms identified in animals are highly relevant to human infants. While direct causal evidence in children is still limited, the convergence of exposure data in humans with mechanistic and outcome data from animal models strongly suggests that early-life exposure to MPs/NPs may pose significant risks for growth, organ development, and long-term health in children. Protecting infants and young children from microplastic exposure should therefore be considered a public health priority. Further research is needed to quantify exposure levels in early life, establish dose-response relationships, and determine whether the adverse developmental outcomes observed in animals translate into similar risks in humans. Such knowledge will be pivotal for risk assessment and the development of regulatory strategies to mitigate potential human health impacts.

## 8 Leeching effect of MP: toxicity generated by MP associated molecules

Plastics consist of a wide range of chemicals, both added and unintentional [190]. Heterogeneous polymers and additive/coating molecules are added depending on the manufacturing needs and the usage of the end product. Also, there are unintentionally added substances (NIAS), like impurities during manufacturing, oligomers and degradation products [191]. These elements could potentially pose their own health risks by leaching from the degraded plastic matrix, which is an indirect consequence of MP pollution [190].

The database of chemicals associated with plastic packaging (CPPdb) has listed 906 chemicals as “likely associated” and an additional 3377 substances as “possibly associated” leeching items. Among the first 906 chemicals, at least 63 have high health hazards [191]. However, a more recent systematic review reported a total of 10,000 MP-related substances, with over 2400 as substances of concern [192]. This huge variation is due to a lack of a standard procedure for measurement. Nevertheless, certain chemicals, such as Bisphenol A (BPA), di(2-ethylhexyl) phthalate (DEHP), and dibutyl phthalate (DBP), have been identified as MP-associated substances that can create health hazards. Animal studies have confirmed that exposure to these chemicals during pregnancy can lead to obesity, reproductive diseases and sperm epimutations in offspring. Moreover, these effects become increasingly pronounced in subsequent generations [193], [194]. Phthalates and BPA are known for endocrine-disrupting functions and are associated with cardiometabolic conditions [195]. More research is necessary to understand the long-term accumulation and its health impacts. Additionally, the synergistic effects of these chemicals with the polymer matrix of the parent plastic and in combination with other substances may lead to more concerning outcomes, which are currently unknown [194].

A second concern regarding the toxicity of MPs arises from the contamination of the plastic matrix during degradation, which is called “load effect”. MPs can absorb a wide range of contaminants from the surrounding environment, including polycyclic aromatic hydrocarbons (PAHs) to microbial agents. Due to their hydrophobic surface, PAHs can easily adhere to MPs. Consequently, MPs can serve as carriers for toxic exposures to various xenobiotics, potentially bypassing typical physiological defences, such as, drug-metabolizing enzymes in the gut and liver, directly affecting the cells and tissues in contact with the internalized MPs [194], [196], [197]. It is worth mentioning that PAHs are genotoxic, carcinogenic and mutagenic, which can significantly affect the health of various life forms, including the vulnerable infants [198].

As a matter of fact, the superior durability and resilience of these MPs ensure their presence and survival in a range of environments, from normal to extreme. This persistence naturally brings them into contact with many pathogenic microbes. A recent hypothesis suggests that in the coming years, humans may encounter microbes previously unknown to cause health hazards due to their lack of human contact, now finding their way by harbouring on MPs. Additionally, other evidence indicates that MPs can promote the evolution of antibiotic resistance and alter host immune responses, indirectly affecting disease transmission conditions [199], [200]. Considering the unique physiology and under developmental sensitivity, the microplastic-associated chemicals have put infants and young children at greater risk for toxicity associated with MPs.

## 9 Conclusion

MP pollution is rapidly emerging as a major environmental threat with potentially serious health consequences. Unlike plastic pollution, which has received significant attention, MP pollution has been largely ignored. This lack of focus is concerning because, unlike established pollutants like heavy metals, there are currently no standardized methods for detecting, controlling, or mitigating MP. If this continues or left unattended, serious consequences may happen in the coming decades.

Key findings from the collected studies indicate that MPs have been detected in prenatal and postnatal samples, including placenta, cord blood, amniotic fluid, breast milk, and infant formula, as well as in early-life environments such as feeding bottles, household dust, play mats, indoor air and even the water, vegetables as well as fruits. Animal studies further suggest that MPs exposure can induce oxidative stress, inflammation, and gut dysbiosis, with potential impacts on retinal, neurological, pulmonary, hepatic, digestive, lymphatic, skeletal, urinary and reproductive systems. Together, these findings raise concerns about early-life vulnerability towards MPs during critical developmental stages.

However, significant limitations of this study is still remained. Human studies are still scarce and they are often based on small sample sizes with heterogeneous methodologies. This makes the comparisons difficult. Standardized protocols for sampling, detection, and quantification are lacking, while dose–response relationships, long-term health effects, and the role of confounding factors remain poorly understood especially in human studies. Majority of the mechanistic evidence is derived from animal models, which may not fully capture human susceptibility or exposure scenarios.

In concise, future research should prioritize on the development and utilization of standardized methods for MPs detection and quantification across biological and environmental matrices. Large-scale, longitudinal human studies are urgently needed to establish exposure–health outcome links, while pre-clinical research is critical to define safe exposure limits and mechanistic pathways. Moreover, effective strategies for monitoring and regulating MPs pollution must be developed to protect the vulnerable populations, especially mothers and young children. Without timely action, the potential long-term health consequences from MP accumulation may eventually become an urgent public health crisis.

## Author contributions

ESST, CKT and YBH contributed to the study conception and design. The manuscript was written by SN, ZXP, RZ, CKT, NAB, SJK and ESST. All authors commented on the manuscript. All authors read and approved the final manuscript.

## CRediT authorship contribution statement

**Stephanie Julia Kosasih:** Writing – review & editing. **Eugenie Sin Sing Tan:** Writing – review & editing, Supervision, Conceptualization. **Zhi Xin Phuna:** Writing – review & editing. **Rahela Zaman:** Writing – original draft. **Sakuntala Nadarasan:** Writing – original draft. **Yu Bin Ho:** Supervision. **Chung Keat Tan:** Writing – review & editing, Supervision. **Normina Ahmad Bustami:** Writing – review & editing, Supervision.

## Consent to publish

Not Applicable.

## Consent to participate

Not Applicable.

## Ethics approval

Not Applicable.

## Funding

This work was financially supported by funds from the Malaysian Ministry of Higher Education under Fundamental Research Grant Scheme via FRGS/1/2023/SKK06/UCSI/02/3 and UCSI University (Malaysia Campus) under the Research Excellence and Innovation Grant (REIG) via REIG-FMHS-2024/005, REIG-FMHS-2024/025 and REIG-FMHS-2025/025.

## Declaration of Competing Interest

The authors declare that they have no known competing financial interests or personal relationships that could have appeared to influence the work reported in this paper.

## Data Availability

Data will be made available on request.
