# Supplementary material for: Microplastics and child health: A scoping review of prenatal and early-life exposure routes and potential health risks
Source: Toxicol Rep. 2025 Oct 14;15:102143. doi: 10.1016/j.toxrep.2025.102143 (PMC12554039; doi:10.1016/j.toxrep.2025.102143)
Supplement: Supplementary file 1 — Supplementary material [file mmc1.docx]

**Appendix 1**

**List of Papers Included**

1. Liu, S., Detection of various microplastics in placentas, meconium, infant feces, breastmilk and infant formula: A pilot prospective study. Sci Total Environ, 2023. 854: p. 158699.

2. Amereh, F., Placental plastics in young women from general population correlate with reduced foetal growth in IUGR pregnancies. Environ Pollut, 2022. 314: p. 120174.

3. Yun, X., et al., Raman-guided exploration of placental microplastic exposure: Unraveling the polymeric tapestry and assessing developmental implications. Journal of Hazardous Materials, 2024. 477: p. 135271.

4. Zhu, M., et al., Microplastic Particles Detected in Fetal Cord Blood, Placenta, and Meconium: A Pilot Study of Nine Mother-Infant Pairs in South China. Toxics, 2024. 12(12).

5. Zhang, M., et al., Microplastic exposure in daily life and the risk of pregnancy-induced hypertension: A study on the association between environmental pollutants and maternal-fetal health outcomes. Journal of Hazardous Materials, 2025. 494: p. 138654.

6. Sun, H., et al., Microplastics in maternal blood, fetal appendages, and umbilical vein blood. Ecotoxicol Environ Saf, 2024. 287: p. 117300.

7. Xue, J., et al., Microplastics in maternal amniotic fluid and their associations with gestational age. Sci Total Environ, 2024. 920: p. 171044.

8. Sun, J., et al., Detection and quantification of various microplastics in human endometrium based on laser direct infrared spectroscopy. Science of The Total Environment, 2024. 906: p. 167760.

9. Zhang, S., et al., Exposure to placental microplastic and placental and umbilical cord blood telomere length. Ecotoxicology and Environmental Safety, 2025. 302: p. 118536.

10. Halfar, J., Microplastics and additives in patients with preterm birth: The first evidence of their presence in both human amniotic fluid and placenta. Chemosphere, 2023. 343: p. 140301.

11. Arshad, N., et al., Microplastic contamination in human breast milk: A disquieting disparity linked to seafood consumption in an economically disadvantaged fishermen community settled along the Karachi coast. Iranian Journal of Fisheries Sciences, 2024. 23(5): p. 727-738.

12. Ragusa, A., Raman Microspectroscopy Detection and Characterisation of Microplastics in Human Breastmilk. Polymers (Basel, 2022. 14(13).

13. Saraluck, A., et al., Detection of Microplastics in Human Breast Milk and Its Association with Changes in Human Milk Bacterial Microbiota. Journal of Clinical Medicine, 2024. 13(14): p. 4029.

14. Zhang, Q., et al., Microplastics in infant milk powder. Environmental Pollution, 2023. 323: p. 121225.

15. Kadac-Czapska, K., et al., Isolation and identification of microplastics in infant formulas – A potential health risk for children. Food Chemistry, 2024. 440: p. 138246.

16. Banica, A.L., et al., Microplastics, Polycyclic Aromatic Hydrocarbons, and Heavy Metals in Milk: Analyses and Induced Health Risk Assessment. Foods, 2024. 13(19): p. 3069.

17. Liu, L., et al., Release of microplastics from breastmilk storage bags and assessment of intake by infants: A preliminary study. Environmental Pollution, 2023. 323: p. 121197.

18. Zhao, L., et al., Microplastics release from infant feeding bottles and milk storage bags. Food Control, 2025. 168: p. 110921.

19. Song, K., et al., Microparticles and microplastics released from daily use of plastic feeding and water bottles and plastic injectors: potential risks to infants and children in China. Environmental Science and Pollution Research, 2021. 28(42): p. 59813-59820.

20. Li, H., et al., Occurrence of microplastics in commercially sold bottled water. Science of The Total Environment, 2023. 867: p. 161553.

21. Xu, Z., et al., Exposure to irregular microplastic shed from baby bottles activates the ROS/NLRP3/Caspase-1 signaling pathway, causing intestinal inflammation. Environment International, 2023. 181: p. 108296.

22. Li, H., et al., The released micro/nano-plastics from plastic containers amplified the toxic response of disinfection by-products in human cells. Food Chemistry, 2025. 470: p. 142636.

23. Su, Y., et al., Steam disinfection releases micro(nano)plastics from silicone-rubber baby teats as examined by optical photothermal infrared microspectroscopy. Nature Nanotechnology, 2022. 17(1): p. 76-85.

24. Ekvall, M.T., et al., Nanoplastics released from daily used silicone and latex products during mechanical breakdown. PLoS One, 2023. 18(9): p. e0289377.

25. Gamage, S. and Y. Mahagamage, Microplastics in personal care products and cosmetics in Sri Lanka. Heliyon, 2024. 10(8): p. e29393.

26. Xue, J., W. Liu, and K. Kannan, Bisphenols, Benzophenones, and Bisphenol A Diglycidyl Ethers in Textiles and Infant Clothing. Environmental Science & Technology, 2017. 51(9): p. 5279-5286.

27. Soltani, N.S., M.P. Taylor, and S.P. Wilson, International quantification of microplastics in indoor dust: prevalence, exposure and risk assessment. Environmental Pollution, 2022. 312: p. 119957.

28. Kashfi, F.S., et al., Occurrence and exposure assessment of microplastics in indoor dusts of buildings with different applications in Bushehr and Shiraz cities, Iran. Science of The Total Environment, 2022. 829: p. 154651.

29. Chakraborty, T.K., et al., Unveiling the hidden pollutants in the indoor environment: Focus on microplastic pollution and its related risks in the educational institutions of megacity, Bangladesh. Environmental Pollution, 2025. 383: p. 126831.

30. Zhu, J., et al., Microplastics in dust from different indoor environments. Science of The Total Environment, 2022. 833: p. 155256.

31. Zhou, J., et al., Seasonal co-occurrence and inhalation risks of microplastics and benzotriazole ultraviolet stabilizers in atmospheric PM2.5 from northern Chinese city. Environment International, 2025. 202: p. 109721.

32. Wu, W., et al., Characterization of airborne microplastics and health risks in high-temperature urban streets: A case study of Nanjing city. Journal of Hazardous Materials, 2025. 496: p. 139431.

33. Prajapati, A., P. Jadhao, and A.R. Kumar, Atmospheric microplastics deposition in a central Indian city: Distribution, characteristics and seasonal variations. Environmental Pollution, 2025. 374: p. 126183.

34. Luo, Y., R. Naidu, and C. Fang, Toy building bricks as a potential source of microplastics and nanoplastics. Journal of Hazardous Materials, 2024. 471: p. 134424.

35. Ou, H., et al., Disinfection inducing release of contaminants from baby play mats: Microplastics and volatile organic compounds. Environmental Pollution, 2025. 366: p. 125497.

36. Han, Z., et al., Occurrence and fate of microplastics from a water source to two different drinking water treatment plants in a megacity in eastern China. Environmental Pollution, 2024. 346: p. 123546.

37. Taghipour, H., et al., Tracking microplastics contamination in drinking water in Zahedan, Iran: From source to consumption taps. Science of The Total Environment, 2023. 872: p. 162121.

38. Ramaremisa, G., H. Tutu, and D. Saad, Detection and characterisation of microplastics in tap water from Gauteng, South Africa. Chemosphere, 2024. 356: p. 141903.

39. Lata, R., et al., Measurement and daily consumption of microplastics in drinking water from a Small Island Developing State—Fiji: from freshwater to groundwater sources. Environmental Monitoring and Assessment, 2025. 197(4): p. 434.

40. Shokunbi, O.S., et al., From source to distribution channel: A baseline study of microplastic occurrence in drinking water in Ogun State, Nigeria. Environmental Monitoring and Assessment, 2025. 197(4): p. 438.

41. Aydın, R.B., et al., Occurrence of Microplastics in Most Consumed Fruits and Vegetables from Turkey and Public Risk Assessment for Consumers. Life (Basel), 2023. 13(8).

42. Altunışık, A., Prevalence of microplastics in commercially sold soft drinks and human risk assessment. Journal of Environmental Management, 2023. 336: p. 117720.

43. Ferrante, M., et al., Microplastics in fillets of Mediterranean seafood. A risk assessment study. Environmental Research, 2022. 204: p. 112247.

44. Bhavsar, P.S., et al., Microplastic contamination in Indian rice: A comprehensive characterization and health risk assessment. Journal of Hazardous Materials, 2024. 480: p. 136208.

45. Koutnik, V.S., et al., Children's playgrounds contain more microplastics than other areas in urban parks. Science of The Total Environment, 2023. 854: p. 158866.

**Appendix 2**

**Scoping Reviews (PRISMA-ScR) Checklist**

| SECTION | ITEM | PRISMA-ScR CHECKLIST ITEM | REPORTED ON PAGE |
| --- | --- | --- | --- |
| TITLE | | | |
| Title | 1 | Identify the report as a scoping review. | 1 |
| ABSTRACT | | | |
| Structured summary | 2 | Provide a structured summary that includes (as applicable): background, objectives, eligibility criteria, sources of evidence, charting methods, results, and conclusions that relate to the review questions and objectives. | 1 |
| INTRODUCTION | | | |
| Rationale | 3 | Describe the rationale for the review in the context of what is already known. Explain why the review questions/objectives lend themselves to a scoping review approach. | 3 |
| Objectives | 4 | Provide an explicit statement of the questions and objectives being addressed with reference to their key elements (e.g., population or participants, concepts, and context) or other relevant key elements used to conceptualize the review questions and/or objectives. | 3 |
| METHODS | | | |
| Protocol and registration | 5 | Indicate whether a review protocol exists; state if and where it can be accessed (e.g., a Web address); and if available, provide registration information, including the registration number. | 5 |
| Eligibility criteria | 6 | Specify characteristics of the sources of evidence used as eligibility criteria (e.g., years considered, language, and publication status), and provide a rationale. | 5 |
| Information sources | 7 | Describe all information sources in the search (e.g., databases with dates of coverage and contact with authors to identify additional sources), as well as the date the most recent search was executed. | 5 |
| Search | 8 | Present the full electronic search strategy for at least 1 database, including any limits used, such that it could be repeated. | 5 |
| Selection of sources of evidence | 9 | State the process for selecting sources of evidence (i.e., screening and eligibility) included in the scoping review. | 5 |
| Data charting process | 10 | Describe the methods of charting data from the included sources of evidence (e.g., calibrated forms or forms that have been tested by the team before their use, and whether data charting was done independently or in duplicate) and any processes for obtaining and confirming data from investigators. | 5 |
| Data items | 11 | List and define all variables for which data were sought and any assumptions and simplifications made. | 5 |
| Critical appraisal of individual sources of evidence | 12 | If done, provide a rationale for conducting a critical appraisal of included sources of evidence; describe the methods used and how this information was used in any data synthesis (if appropriate). | 5 |
| Synthesis of results | 13 | Describe the methods of handling and summarizing the data that were charted. | 5 |
| RESULTS | | | |
| Selection of sources of evidence | 14 | Give numbers of sources of evidence screened, assessed for eligibility, and included in the review, with reasons for exclusions at each stage, ideally using a flow diagram. | 6 |
| Characteristics of sources of evidence | 15 | For each source of evidence, present characteristics for which data were charted and provide the citations. | 6 |
| Critical appraisal within sources of evidence | 16 | If done, present data on critical appraisal of included sources of evidence (see item 12). | 6 |
| Results of individual sources of evidence | 17 | For each included source of evidence, present the relevant data that were charted that relate to the review questions and objectives. | 6 |
| Synthesis of results | 18 | Summarize and/or present the charting results as they relate to the review questions and objectives. | 6 |
| DISCUSSION | | | |
| Summary of evidence | 19 | Summarize the main results (including an overview of concepts, themes, and types of evidence available), link to the review questions and objectives, and consider the relevance to key groups. | 7 - 39 |
| Limitations | 20 | Discuss the limitations of the scoping review process. | 56 |
| Conclusions | 21 | Provide a general interpretation of the results with respect to the review questions and objectives, as well as potential implications and/or next steps. | 56 |
| FUNDING | | | |
| Funding | 22 | Describe sources of funding for the included sources of evidence, as well as sources of funding for the scoping review. Describe the role of the funders of the scoping review. | 57 |
